# Supplementary material for: Development of an NLR-ID Toolkit and Identification of Novel Disease-Resistance Genes in Soybean
Source: Plants (Basel). 2024 Feb 28;13(5):668. doi: 10.3390/plants13050668 (PMC10934728; doi:10.3390/plants13050668)
Supplement: Supplementary file 1 [file plants-13-00668-s001.zip › plants-2870959-supplementary.pdf]

Table S1 Sequence information of 210 TNL-like proteins.

Number	Gene ID	Length（aa)	Primary accession	Type
1	*GLYMA_01G032400*	1453 aa	K7K1I4	TNLX
2	*GLYMA_01G032900*	956 aa	K7K1J3	TNL
3	*GLYMA_01G033200*	1417 aa	A0A0R0LCQ5	TNL
4	*GLYMA_01G033300*	1201 aa	A0A0R0L5W8	TNL
5	*GLYMA_01G039000*	1379 aa	I1J5H4	TNL
6	*GLYMA_01G046900*	1036 aa	A0A0R0L6J4	TNL
7	*GLYMA_01G112200*	1227 aa	K7K360	TNLX
8	*GLYMA_01G112300*	1258 aa	K7K361	TNL
9	*GLYMA_01G119800*	175 aa	K7K3C5	T
10	*GLYMA_01G125300*	1063 aa	K7K3H1	TNL
11	*GLYMA_01G125400*	1156 aa	K7K3H2	TNL
12	*GLYMA_01G220900*	999 aa	K7K584	T
13	*GLYMA_02G023600*	169 aa	A0A0R0KQA9	T
14	*GLYMA_02G023700*	263 aa	K7K612	T
15	*GLYMA_02G023800*	257 aa	C6ZS34	T
16	*GLYMA_02G023900*	263 aa	C6ZS33	T
17	*GLYMA_02G024000*	257 aa	C6THX9	TX
18	*GLYMA_02G033000*	787 aa	A0A0R0KY80	TNL
19	*GLYMA_02G041800*	784 aa	K7K6E2	TNX
20	*GLYMA_02G077000*	426 aa	A0A0R0L0H1	TNX
21	*GLYMA_02G193400*	427 aa	K7K9I6	T
22	*GLYMA_02G222500*	745 aa	A0A0R0KZP3	TNLX
23	*GLYMA_02G268900*	1177 aa	K7KB13	TNL
24	*GLYMA_02G284000*	101 aa	K7KBB1	T
25	*GLYMA_02G284100*	1548 aa	K7KBB2	TNL
26	*GLYMA_02G284200*	1683 aa	A0A0R0L2V4	TNL
27	*GLYMA_02G290300*	380 aa	I1JJ81	TTX
28	*GLYMA_02G290400*	375 aa	C6TIX3	TTX
29	*GLYMA_03G047700*	829 aa	A0A0R0KP92	TNLX
30	*GLYMA_03G047900*	1049 aa	A0A0R0KES8	TNL
31	*GLYMA_03G048500*	1012 aa	A0A0R0KET5	TNL
32	*GLYMA_03G048700*	531aa	A0A0R0KEW1	TN
33	*GLYMA_03G048800*	185 aa	K7KCX0	T
34	*GLYMA_03G052800*	1444 aa	K7KCZ9	TNLX
35	*GLYMA_03G053300*	191 aa	K7KD03	T
36	*GLYMA_03G053500*	758 aa	A0A0R0KF26	TN
37	*GLYMA_03G054100*	887 aa	A0A0R0KLY1	TNL
38	*GLYMA_03G054600*	316 aa	A0A0R0KFL6	TX
39	*GLYMA_03G075300*	1128 aa	K7KDI2	TNL
40	*GLYMA_03G077400*	1127 aa	K7KDG9	TNLX
41	*GLYMA_03G087500*	1084 aa	I1JM77	TNLX
42	*GLYMA_03G087600*	772 aa	A0A0R0KGG0	TNL

(continued table)

Number	Gene ID	Length（aa）	Primary accession	Type
43	*GLYMA_03G088000*	905 aa	K7KDW3	TNL
44	*GLYMA_03G088100*	1081 aa	K7KDW5	TNL
45	*GLYMA_04G219600*	464 aa	A0A0R0KB65	TNL
46	*GLYMA_05G165800*	1355 aa	K7KQM9	TNLX
47	*GLYMA_06G146200*	222 aa	K7KV37	TX
48	*GLYMA_06G205100*	397 aa	A0A0R0JJM6	TN
49	*GLYMA_06G216900*	112 aa	A0A0R0JKE6	T
50	*GLYMA_06G248700*	204 aa	I1KDZ9	T
51	*GLYMA_06G256000*	1158 aa	A0A0R0JTT7	TNL
52	*GLYMA_06G256100*	180 aa	K7KXC7	T
53	*GLYMA_06G259100*	1091 aa	I1KE91	TNL
54	*GLYMA_06G259400*	1099 aa	I1KE93	TNL
55	*GLYMA_06G259800*	1202 aa	A0A0R0JLQ6	TNL
56	*GLYMA_06G260100*	1098 aa	I1KE99	TNL
57	*GLYMA_06G260500*	572 aa	A0A0R0JLQ8	TL
58	*GLYMA_06G261400*	1523 aa	A0A0R0JT19	XTNL
59	*GLYMA_06G261500*	1110 aa	I1KEB4	TNLX
60	*GLYMA_06G263500*	1143 aa	K7KXJ1	TNL
61	*GLYMA_06G263700*	380 aa	K7KXJ4	TT
62	*GLYMA_06G263900*	1322 aa	A0A0R0JM06	TNL
63	*GLYMA_06G264100*	336 aa	A0A0R0JXY0	TT
64	*GLYMA_06G264300*	522 aa	A0A0R0JLW9	TNX
65	*GLYMA_06G265000*	1310 aa	A0A0R0JS96	TNL
66	*GLYMA_06G265200*	266 aa	K7KXK7	TT
67	*GLYMA_06G265400*	1296 aa	A0A0R0JM20	TNLX
68	*GLYMA_06G267300*	636 aa	Q8H6S7	TNX
69	*GLYMA_06G267400*	1238 aa	K7KXM9	TNTT
70	*GLYMA_06G268500*	412 aa	A0A0R0JT99	TTX
71	*GLYMA_06G268600*	641 aa	I1KEG1	TNX
72	*GLYMA_06G268700*	1387 aa	A0A0R0JM07	TTTNL
73	*GLYMA_06G285500*	1084 aa	I1KEV5	TNL
74	*GLYMA_06G310000*	1464 aa	K7KYE4	TNLX
75	*GLYMA_07G007900*	110 aa	A0A0R0J4I4	T
76	*GLYMA_07G037000*	1090 aa	K7KZI3	TNLX
77	*GLYMA_07G067900*	905 aa	A0A0R0J6E0	TNL
78	*GLYMA_07G123000*	1171 aa	K7L0Z9	TNL
79	*GLYMA_07G193000*	185 aa	I1KLG4	T
80	*GLYMA_08G159200*	125 aa	A0A0R0IMV1	T
81	*GLYMA_08G192900*	1137 aa	K7L7M1	TNL
82	*GLYMA_08G293600*	1430 aa	K7L9P4	TNL
83	*GLYMA_08G295000*	265 aa	K7L9Q3	T
84	*GLYMA_08G295100*	150 aa	A0A0R0J0J4	T

（continued table）

Number	Gene ID	Length（aa）	Primary accession	Type
85	*GLYMA_08G295400*	128 aa	A0A0R0J042	TX
86	*GLYMA_08G301200*	1231 aa	K7L9U4	TNLX
87	*GLYMA_08G303600*	362 aa	K7L9W1	TN
88	*GLYMA_08G303700*	882 aa	K7L9W3	TNL
89	*GLYMA_09G056400*	1072 aa	I1L1A5	TNLD
90	*GLYMA_09G056500*	67 aa	K7LBZ9	T
91	*GLYMA_09G057000*	1036 aa	K7LC01	TNL
92	*GLYMA_09G075500*	1055 aa	A0A0R0IEZ9	TNL
93	*GLYMA_09G134400*	441 aa	A0A0R0IDT0	TLT
94	*GLYMA_09G161400*	1390 aa	K7LE88	TNXLD
95	*GLYMA_09G164800*	459 aa	A0A0R0IFK2	TND
96	*GLYMA_09G202800*	1008 aa	A0A0R0IAY8	TNL
97	*GLYMA_10G184300*	1220 aa	A0A0R0HV98	TNL
98	*GLYMA_10G184400*	1030 aa	A0A0R0HVF4	TNX
99	*GLYMA_11G022700*	1008 aa	K7LMN0	T
100	*GLYMA_11G153000*	1085 aa	K7LQJ9	TNL
101	*GLYMA_11G160500*	636 aa	K7KPR3	TTNL
102	*GLYMA_12G027100*	1163 aa	K7LSQ4	TNL
103	*GLYMA_12G132000*	1076 aa	K7LUI6	TNL
104	*GLYMA_12G132200*	1087 aa	K7LUI7	TNL
105	*GLYMA_12G132400*	411 aa	A0A0R0H572	TN
106	*GLYMA_12G133000*	620 aa	K7LUJ2	TL
107	*GLYMA_12G135600*	1141 aa	I1LSM9	TNL
108	*GLYMA_12G137900*	902 aa	A0A0R0HDM9	TNL
109	*GLYMA_12G138400*	685 aa	A0A0R0HEA3	TNL
110	*GLYMA_12G138500*	234 aa	A0A0R0H5P2	TND
111	*GLYMA_12G163000*	522 aa	A0A0R0HG01	TNL
112	*GLYMA_12G212500*	1190 aa	K7LW75	TNL
113	*GLYMA_12G221600*	200 aa	I1LUY0	T
114	*GLYMA_12G239200*	919 aa	K7LWN3	TNL
115	*GLYMA_12G239700*	1481 aa	K7LWN5	TNL
116	*GLYMA_12G239800*	1368 aa	A0A0R0HAA3	TNL
117	*GLYMA_12G240100*	1014 aa	K7LWN7	TNL
118	*GLYMA_13G028100*	1036 aa	K7LYT2	TNL
119	*GLYMA_13G076200*	979 aa	I1LWA0	TNL
120	*GLYMA_13G078200*	576 aa	A0A0R0GJV2	TNL
121	*GLYMA_13G194700*	1095 aa	I1M0P9	TNL
122	*GLYMA_13G194800*	490 aa	I1M0Q0	TND
123	*GLYMA_13G194900*	530 aa	I1M0S0	TNX
124	*GLYMA_13G195100*	1095 aa	A0A0R0GQV9	TNL
125	*GLYMA_13G242900*	106 aa	K7M1M8	T
126	*GLYMA_13G243000*	193 aa	I1M289	T

（continued table）

Number	Gene ID	Length（aa）	Primary accession	Type
127	*GLYMA_13G279700*	200 aa	A0A0R0GW30	T
128	*GLYMA_14G024400*	337 aa	C6ZS32	TT
129	*GLYMA_14G024500*	370 aa	A0A0R4J566	TT
130	*GLYMA_14G048600*	1127 aa	K7M4Z6	TNL
131	*GLYMA_14G151100*	1167 aa	K7M6W8	TNL
132	*GLYMA_14G152600*	128 aa	A0A0R0GKC9	T
133	*GLYMA_15G024800*	1158 aa	I1MD01	TNL
134	*GLYMA_15G070400*	182 aa	I1MEE1	T
135	*GLYMA_15G070500*	181 aa	K7MA27	T
136	*GLYMA_15G152200*	1161 aa	A0A0R0G1P5	TNLX
137	*GLYMA_15G152300*	87 aa	A0A0R0G9S6	T
138	*GLYMA_15G152400*	1149 aa	A0A0R0G9U0	TNL
139	*GLYMA_15G160900*	1106 aa	K7MBP2	TNL
140	*GLYMA_15G162700*	929 aa	A0A0R0G1P9	TNL
141	*GLYMA_15G232400*	498 aa	K7MDC7	TND
142	*GLYMA_15G232600*	494 aa	A0A0R0G4X0	TN
143	*GLYMA_15G232700*	522 aa	K7MDD3	TN
144	*GLYMA_16G006400*	1054 aa	K7MEG0	TNLX
145	*GLYMA_16G033900*	1162 aa	A0A0R0FKV4	TNL
146	*GLYMA_16G085400*	981 aa	A0A0R0FN48	TNL
147	*GLYMA_16G085700*	1059 aa	I1MM77	TNLX
148	*GLYMA_16G085900*	1113 aa	I1MM78	TNL
149	*GLYMA_16G086500*	987 aa	K7MG05	TNL
150	*GLYMA_16G086700*	1036 aa	A0A0R0FN84	TNL
151	*GLYMA_16G087100*	1041 aa	K7MG09	TNLX
152	*GLYMA_16G118600*	797 aa	I1MMX8	TN
153	*GLYMA_16G127900*	1422 aa	A0A0R0G1J4	TNLX
154	*GLYMA_16G128000*	997 aa	A0A0R0FQ34	TNL
155	*GLYMA_16G135200*	1058 aa	K7MH68	TNL
156	*GLYMA_16G135500*	1047 aa	I1MNC5	TNL
157	*GLYMA_16G135900*	376 aa	I1MNC7	TNX
158	*GLYMA_16G136000*	1055 aa	A0A0R0FQC6	TNL
159	*GLYMA_16G136200*	956 aa	A0A0R0FQD0	TNL
160	*GLYMA_16G136600*	1403 aa	K7MH77	TNL
161	*GLYMA_16G136900*	907 aa	A0A0R0FQD6	TNL
162	*GLYMA_16G137200*	423 aa	I1MND4	TN
163	*GLYMA_16G137300*	1029 aa	I1MND5	TNL
164	*GLYMA_16G137600*	1079 aa	A0A0R0FZ27	TNL
165	*GLYMA_16G147000*	709 aa	A0A0R0G2F8	TNL
166	*GLYMA_16G147400*	732 aa	A0A0R0FZL1	TNL
167	*GLYMA_16G159100*	1149 aa	K7MHM2	TNL
168	*GLYMA_16G159200*	272 aa	A0A0R0G0Q0	TN

(continued table)

Number	Gene ID	Length（aa）	Primary accession	Type
169	*GLYMA_16G159400*	176 aa	K7MHM5	T
170	*GLYMA_16G159600*	1079 aa	A0A0R0G1Y1	TNL
171	*GLYMA_16G159700*	1040 aa	K7MHM7	TNL
172	*GLYMA_16G198900*	1087 aa	A0A0R0FT41	TNL
173	*GLYMA_16G209000*	164 aa	A0A0R0G203	T
174	*GLYMA_16G210600*	1420 aa	I1MQE7	TNLX
175	*GLYMA_16G210800*	1432 aa	K7MIT8	TNLX
176	*GLYMA_16G211400*	1288 aa	A0A0R0G194	TNL
177	*GLYMA_16G212300*	1051 aa	D6PT14	TNL
178	*GLYMA_16G213700*	1088 aa	A0A0R0FTG2	TNL
179	*GLYMA_16G213800*	1098 aa	A0A0R0FTF9	TNL
180	*GLYMA_16G213900*	1097 aa	A0A0R0FTC3	TNL
181	*GLYMA_16G214000*	966 aa	A0A0R0FT79	TNL
182	*GLYMA_16G214100*	1103 aa	I1MQI3	TNL
183	*GLYMA_16G214200*	641 aa	Q84ZU9	TNX
184	*GLYMA_16G214300*	670 aa	A0A0R0FTY7	TNX
185	*GLYMA_16G214500*	264 aa	Q84ZV1	TX
186	*GLYMA_16G214600*	1044 aa	A0A0R0FTH7	TNL
187	*GLYMA_16G214700*	77 aa	Q84ZV4	T
188	*GLYMA_16G214800*	305 aa	K7MIY3	TX
189	*GLYMA_16G214900*	923 aa	A0A0R0FTD8	TNL
190	*GLYMA_16G215000*	1105 aa	A0A0R0G4T7	TNL
191	*GLYMA_16G215100*	353 aa	K7MIY7	TN
192	*GLYMA_16G215200*	68 aa	K7MIY8	T
193	*GLYMA_16G215400*	393 aa	K7MIZ0	TN
194	*GLYMA_17G197100*	211 aa	K7MMQ0	T
195	*GLYMA_18G127800*	136 aa	A0A0R0F079	T
196	*GLYMA_18G127900*	299 aa	C6ZS22	T
197	*GLYMA_18G128000*	171 aa	K7MRQ4	T
198	*GLYMA_19G022700*	1073 aa	I1N650	TNL
199	*GLYMA_19G054700*	1080 aa	I1N6X6	TNL
200	*GLYMA_19G054800*	723 aa	A0A0R0EJF1	TNL
201	*GLYMA_19G054900*	1124 aa	K7MWR8	TNL
202	*GLYMA_19G055000*	1119 aa	K7MWR9	TNL
203	*GLYMA_20G020200*	1344 aa	K7N0U7	TNL
204	*GLYMA_20G020500*	214 aa	A0A0R0E5K4	T
205	*GLYMA_20G048800*	1114 aa	A0A0R0E807	TNL
206	*GLYMA_20G061300*	1033 aa	K7N1C6	TNL
207	*GLYMA_U005000*	199 aa	K7KCX9	T
208	*GLYMA_U008300*	1070 aa	A0A0R0E307	TNL
209	*GLYMA_U035400*	825 aa	A0A0R0EE48	TNL
210	*GLYMA_U035700*	1043 aa	A0A0R0E2U1	TNL

Table S2 Soybean *Gm-TNL-ID* genes information.

Gene names	Gene ID	Gene Description	Integrated domain
Gm-TNL-ID1	*GLYMA_13G194700*	TMV resistance protein N like	DUF4571
Gm-TNL-ID2	*GLYMA_06G265000*	disease resistance protein RPV1 like	DUF4277
Gm-TNL-ID3	*GLYMA_03G087500*		DUF2207
Gm-TNL-ID4	*GLYMA_12G240100*	disease resistance protein RUN1 like	AAA22
Gm-TNL-ID5	*GLYMA_15G152400*		AAA22
Gm-TNL-ID6	*GLYMA_06G265400*	LRR domain-containing protein	DUF3760
Gm-TNL-ID7	*GLYMA_16G087100*	disease resistance protein RUN1 like	ATPase2
Gm-TNL-ID8	*GLYMA_13G078200*		FBD
Gm-TNL-ID9	*GLYMA_06G310000*	TMV resistance protein N like	AnfG_VnfG
Gm-TNL-ID10	*GLYMA_09G056400*	disease resistance protein RPV1 like	DUF3557
Gm-TNL-ID11	*GLYMA_16G210800*	candidate disease-resistance protein	zf-RVT, RVT3
Gm-TNL-ID12	*GLYMA_16G127900*		zf-RVT, RVT3
Gm-TNL-ID13	*GLYMA_09G161400*	TMV resistance protein N like	zf-RVT, RVT3
Gm-TNL-ID14	*GLYMA_16G210600*	TMV resistance protein N like	zf-RVT, RVT3
Gm-TNL-ID15	*GLYMA_08G301200*	TMV resistance protein N like	zf-RVT
Gm-TNL-ID16	*GLYMA_06G285500*	disease resistance protein RUN1 like	Zinc_ribbon 13
Gm-TNL-ID17	*GLYMA_05G165800*		WRKY
Gm-TNL-ID18	*GLYMA_03G075300*	disease resistance protein RPV1 like	RNA_helicase
Gm-TNL-ID19	*GLYMA_03G077400*	disease resistance protein RPV1 like	NfeD
Gm-TNL-ID20	*GLYMA_07G123000*	disease resistance protein RPV1 like	RICTOR_M
Gm-TNL-ID21	*GLYMA_16G085700*	disease resistance protein RUN1 like	Pox_RNA_Pol _19
Gm-TNL-ID22	*GLYMA_03G047900*		Phage_rep_org_N
Gm-TNL-ID23	*GLYMA_16G006400*	disease resistance protein RPV1 like	NTPase1
Gm-TNL-ID24	*GLYMA_07G037000*	disease resistance protein RPV1 lke	NTPase1
Gm-TNL-ID25	*GLYMA_06G267300*	KR3 disease resistance protein KR3	MotA_activ
Gm-TNL-ID26	*GLYMA_16G213700*		MotA_activ
Gm-TNL-ID27	*GLYMA_19G022700*	TMV resistance protein N like	MotA_activ
Gm-TNL-ID28	*GLYMA_16G159600*		MotA_activ
Gm-TNL-ID29	*GLYMA_19G054900*	KR1 resistance protein KR1	MotA_activ
Gm-TNL-ID30	*GLYMA_19G054700*	disease resistance protein RPV1 like	MotA_activ
Gm-TNL-ID31	*GLYMA_16G147400*		MotA_activ
Gm-TNL-ID32	*GLYMA_16G137300*	disease resistance protein RUN1 like	MotA_activ
Gm-TNL-ID33	*GLYMA_16G136000*		MotA_activ
Gm-TNL-ID34	*GLYMA_16G136200*		MotA_activ
Gm-TNL-ID35	*GLYMA_16G135500*	TMV resistance protein N ilke	MotA_activ
Gm-TNL-ID36	*GLYMA_16G135200*	Disease resistance protein RUN1 like	MotA_activ
Gm-TNL-ID37	*GLYMA_03G052800*	Disease resistance protein RUN1 like	Hirudin
Gm-TNL-ID38	*GLYMA_01G032400*	TMV resistance protein N like	HAUS_augmin3
Gm-TNL-ID39	*GLYMA_14G151100*	Disease resistance protein RPV1 like	NACHT
Gm-TNL-ID40	*GLYMA_16G085400*		NACHT
Gm-TNL-ID41	*GLYMA_15G152200*	disease resistance-like protein DSC1 like	FNIP

Table S3 Identification of proteins interacting with SRZ4.

Gene ID	Annotations
GLYMA_06G029400	*Glycine max* 26S proteasome regulatory subunit 10B homolog A, mRNA
GLYMA_11G021400	*Glycine max* 2-C-methyl-D-erythritol 2,4-cyclodiphosphate synthase, chloroplastic-like
GLYMA_10G078400	*Glycine max* 2-cys peroxiredoxin, mRNA
GLYMA_20G082800	*Glycine max* 31 kDa ribonucleoprotein, chloroplastic-like, mRNA
GLYMA_11G078700	*Glycine max* 3-dehydroquinate synthase, transcript variant X1, mRNA
GLYMA_19G254600	*Glycine max* anthocyanidin reductase, mRNA
GLYMA_08G036400	*Glycine max* auxin-responsive protein IAA27-like , transcript variant X1, mRNA
GLYMA_04G082900	*Glycine max* casein kinase 1-like protein 4
GLYMA_05G128000	*Glycine max* chlorophyll a-b-binding protein (CAB3), mRNA
GLYMA_09G154700	*Glycine max* chlorophyll a-b binding protein CP26
GLYMA_06G194900	*Glycine max* chlorophyll a-b binding protein P4, chloroplastic, mRNA
GLYMA_05G059200	*Glycine max* Cwf15/Cwc15 cell cycle control family protein, mRNA
GLYMA_08G241600	*Glycine max* cyclic nucleotide-gated ion channel 2, transcript variant X2, mRNA
GLYMA_11G113500	*Glycine max* cysteine protease RD19A, mRNA
GLYMA_18G154900	*Glycine max* cytochrome b5, mRNA
GLYMA_11G213600	*Glycine max* cytochrome c oxidase assembly protein COX11, mitochondrial-like, mRNA
GLYMA_13G237000	*Glycine max* dormancy-associated protein 1-like, transcript variant 1, mRNA
GLYMA_11G110000	*Glycine max* epimerase family protein slr1223-like, mRNA; nuclear gene for chloroplast product
GLYMA_01G028800	*Glycine max* F-box/kelch-repeat protein At1g23390, mRNA
GLYMA_11G111400	*Glycine max* fructose-bisphosphate aldolase 1, chloroplastic, mRNA
GLYMA_17G100700	*Glycine max* geranylgeranyl diphosphate reductase, chloroplastic, mRNA
GLYMA_06G015900	*Glycine max* glyceraldehyde-3-phosphate dehydrogenase B subunit, transcript variant 2, mRNA
GLYMA_05G180600	*Glycine max* inositol-3-phosphate synthase , mRNA
GLYMA_07G185400	*Glycine max* malate dehydrogenase, glyoxysomal-like, mRNA
GLYMA_17G063000	*Glycine max* NADPH--cytochrome P450 reductase 2, mRNA
GLYMA_11G061300	*Glycine max* oxygen-evolving enhancer protein 1, chloroplastic-like
GLYMA_08G124900	*Glycine max* PDZ and TPR domain-containing protein, mRNA
GLYMA_01G214300	*Glycine max* PGR5-like protein 1A, chloroplastic, transcript variant X2, misc_RNA
GLYMA_10G222600	*Glycine max* phytosulfokine receptor 2, mRNA
GLYMA_04G020300	*Glycine max* plastocyanin, mRNA
GLYMA_11G014900	*Glycine max* probable acyl-activating enzyme 1, peroxisomal, mRNA
GLYMA_10G212200	*Glycine max* probable ubiquitin-conjugating enzyme E2 C, mRNA
GLYMA_01G077600	*Glycine max* protease Do-like 9, transcript variant X2, mRNA
GLYMA_19G260600	*Glycine max* protein curvature thylakoid 1B-like, mRNA
GLYMA_18G213500	*Glycine max* protein fatty acid export 3, chloroplastic, transcript variant X2, mRNA
GLYMA_09G043800	*Glycine max* protein pleiotropic regulatory locus 1, transcript variant X2, mRNA
GLYMA_09G227100	*Glycine max* protein reticulata-related 4, chloroplastic, mRNA
GLYMA_08G309300	*Glycine max* ribose-phosphate pyrophosphokinase 4
GLYMA_16G089000	*Glycine max* putative 1-deoxy-D-xylulose 5-phosphate reductoiso-merase (DXR2)
GLYMA_03G202900	*Glycine max* putative chloroplast RNA-binding protein 33, mRNA; nuclear gene for chloroplast product
GLYMA_17G073300	*Glycine max* signal recognition particle receptor subunit alpha, mRNA

(continued table)

Accession number	Annotations
GLYMA_02G195400	*Glycine max* syntaxin-121, mRNA
GLYMA_20G001600	*Glycine max* transcription factor TCP7, mRNA
GLYMA_08G206000	*Glycine max* translation machinery associated TMA7 superfamily protein, mRNA
GLYMA_19G127700	*Glycine max* tubulin beta-4 chain, mRNA
GLYMA_16G037700	*Glycine max* U5 small nuclear ribonucleoprotein 40 kDa protein-like, mRNA
GLYMA_09G023700	*Glycine max* ubiquitin-40S ribosomal protein S27a
GLYMA_08G112000	*Glycine max* UBX domain-containing protein 1, transcript variant X2, mRNA

Table S4 Primers used in this study.

Primer name	Sequence（5′-3′）	Purpose
Gm-TNL-ID1-F	ATGTCGAAGGCAGTTTCAGAATC	ORF sequence
Gm-TNL-ID1-R	TTATAAGATGGTGGGTTTATAAGGATC	cloned in pCE2
Gm-TNL-ID2-F	ATGGCTTCTAAGGCCATCATCC	
Gm-TNL-ID2-R	CTACTGCTGCTTCCACTGT	
Gm-TNL-ID3-F	ATGGAATTAGAGTCTTCTTC	
Gm-TNL-ID3-R	TTACAATTCTTCTCTGCGTGGAAATAC	
Gm-TNL-ID5-F	ATGTTGTATTTTTTTGTCTA	
Gm-TNL-ID5-R	TTAAACATGGAGCGAGTCATG	
Gm-TNL-ID7-F	ATGTCATCTTCTTCATTCTCAAC	
Gm-TNL-ID7-R	CTACTGCTCTTCATTTTTACTTGATA	
Gm-TNL-ID8-F	ATGGAATGCAAGAAACAAGGAG	
Gm-TNL-ID8-R	TCAATCGTTGAACTTACTTAACACTG	
Gm-TNL-ID9-F	ATGGCTGTGATGCTGTGTGGAG	
Gm-TNL-ID9-R	TTAATCAAACCCTTCATGCCTTAAG	
Gm-TNL-ID10-F	ATGTCCAACAAAGCTGCTCC	
Gm-TNL-ID10-R	TCAGTCACTGTCATGCAATTC	
Gm-TNL-ID11-F	ATGGCTTGGGGATCACG	
Gm-TNL-ID11-R	TTATATGTATTCACTAACAAAAGGTTGG	
Gm-TNL-ID12-F	ATGGCTACGGGATCACCT	
Gm-TNL-ID12-R	TTACGTAACATGTAACATGTATCTCTC	
Gm-TNL-ID14-F	ATGGCTTGGGGATCAAGTTC	
Gm-TNL-ID14-R	TTATCTGTATTCTGAAATATAAGGTTGGC	
Gm-TNL-ID15-F	ATGACACAGCAATCATGTTCC	
Gm-TNL-ID15-R	TTACCTTAAAATCAATGGATTGACAG	
Gm-TNL-ID16-F	ATGGCTTCCAACAACAACAG	
Gm-TNL-ID16-R	CTAGTTTCCATCTTCCAAAGAAAGG	
Gm-TNL-ID17-F	ATGGCTTCCTCGGCCTT	
Gm-TNL-ID17-R	TTATAACTTGCAGGATTGACTTGC	
Gm-TNL-ID18-F	ATGCCAACAGAGATTGCAATG	
Gm-TNL-ID18-R	TCAAGTTTGACCTAACATTCTTTCAC	
Gm-TNL-ID19-F	ATGCCTTCCGATCGTGAAAT	
Gm-TNL-ID19-R	TCATTCAATTTTTCTTCTTTTCTGTTTTT	
Gm-TNL-ID21-F	ATGGAATTCGTGGCTTCTTC	
Gm-TNL-ID21-R	CTACTGCTCTTGAATCTTGCTCG	
Gm-TNL-ID23-F	ATGGCTTTCCAAGTTTCTCATA	
Gm-TNL-ID23-R	TCACCCATTCCACACGGA	
Gm-TNL-ID24-F	ATGCCATATTCTTATTCGTTCCG	
Gm-TNL-ID24-R	TTATTCTACTAACCTTCTAAAATAAATCCTAGA	
Gm-TNL-ID25-F	ATGAAGTTTGGTTCTATATTCCAACAG	
Gm-TNL-ID25-R	CTATTCCAAGTCGCGTATGGC	

(continued table)

Primer name	Sequence（5′-3′）	Purpose
Gm-TNL-ID26-F	ATGGCTGCGACAACACG	
Gm-TNL-ID26-R	CTATGGTTTGTAGTCTGATGCAG	
Gm-TNL-ID28-F	ATGTCCTCCTCCTCCATTAGCT	
Gm-TNL-ID28-R	CTAAAAAATAACCAAATGATGACTTATTTGG	
Gm-TNL-ID29-F	ATGGCTAAGCAATCATCCTCAT	
Gm-TNL-ID29-R	TCATTGATTGTTGCGGCG	
Gm-TNL-ID30-F	ATGGCTATGCAATCATCCTCA	
Gm-TNL-ID30-R	TTATGATTCTGAACTATTGAAATCATCAA	
Gm-TNL-ID35-F	ATGGCTGTGCGATCATTCTC	
Gm-TNL-ID35-R	TTATGAACTAATGAGATCATCATTTAATTTTTT	
Gm-TNL-ID36-F	ATGGCTCTGCGATCATTCT	
Gm-TNL-ID36-R	CTACAAATTGTGTCTCAACAACTTC	
Gm-TNL-ID39-F	ATGCCCTCACTATTGCGAG	
Gm-TNL-ID39-R	TTACCAGAAGTCACACATTACAAT	
BD-ID1^{X}-F	CatatggccatggaggccgaattcTACCTCGAGAAGGAGGTTTTTC	ID^{X} sequence cloned
BD-ID1^{X}-R	tagttatgcggccgctgcaggtcgacTTATAAGATGGTGGGTTTATAAGGATC	in pGBKT7
BD-ID2^{X}-F	catatggccatggaggccgaattcAATGACAGGGAAATATTTTTAGATATTGC	
BD-ID2^{X}-R	tagttatgcggccgctgcaggtcgacCTACTGCTGCTTCCACTGT	
BD-ID3^{X}-F	catatggccatggaggccgaattcAAGGATATATTCCTTGATGTGTGT	
BD-ID3^{X}-R	tagttatgcggccgctgcaggtcgacTTACAATTCTTCTCTGCGTGGAAATAC	
BD-ID4^{X}-F	catatggccatggaggccgaattcGAAGATGAAAAGGGAATTTTCCTTG	
BD-ID4^{X}-R	tagttatgcggccgctgcaggtcgacTTATACAACTTGATAATACAAGCAACC	
BD-ID5^{X}-F	catatggccatggaggccgaattcCAGATTTTTCTAGATTTGGCATGTTT	
BD-ID5^{X}-R	tagttatgcggccgctgcaggtcgacTTAAACATGGAGCGAGTCATG	
BD-ID7^{X}-F	catatggccatggaggccgaattcGAAAAGGATATATTTCTTGATATATGTTGTT	
BD-ID7^{X}-R	tagttatgcggccgctgcaggtcgacCTACTGCTCTTCATTTTTACTTGATA	
BD-ID8^{X}-F	catatggccatggaggccgaattcTTTTCTGCAGATATAGGGACAAGAAG	
BD-ID8^{X}-R	tagttatgcggccgctgcaggtcgacTCAATCGTTGAACTTACTTAACACTG	
BD-ID9^{X}-F	catatggccatggaggccgaattcATTCCCAACAAAGAGGTTCAGAA	
BD-ID9^{X}-R	tagttatgcggccgctgcaggtcgacTTAATCAAACCCTTCATGCCTTAAG	
BD-ID10^{X}-F	catatggccatggaggccgaattcGATCGTAAAGAGCAACAAATTTTTCT	
BD-ID10^{X}-R	tagttatgcggccgctgcaggtcgacTCAGTCACTGTCATGCAATTC	
BD-ID11^{X}-F	catatggccatggaggccgaattcGAAGAAGAGAAGAAAGTTTTTCTTGAC	
BD-ID11^{X}-R	tagttatgcggccgctgcaggtcgacTTATATGTATTCACTAACAAAAGGTTGG	
BD-ID12^{X}-F	catatggccatggaggccgaattcATGGCTACGGGATCACCT	
BD-ID12^{X}-R	tagttatgcggccgctgcaggtcgacTTACGTAACATGTAACATGTATCTCTC	
BD-ID13^{X}-F	catatggccatggaggccgaattcGAAGAGAAAAGTGTTTTTCTTGACC	
BD-ID13^{X}-R	tagttatgcggccgctgcaggtcgacTCAAACATCTACAAAAGGTGCAC	
BD-ID14^{X}-F	catatggccatggaggccgaattcAAGGAAATTCTAGATGTACTTACAGTA	
BD-ID14^{X}-R	tagttatgcggccgctgcaggtcgacTTATCTGTATTCTGAAATATAAGGTTGGC	
BD-ID15^{X}-F	catatggccatggaggccgaattcAATGAGAAGGAAGTTTTCCTTGAC	
BD-ID15^{X}-R	tagttatgcggccgctgcaggtcgacTTACCTTAAAATCAATGGATTGACAG	

(continued table)

Primer name	Sequence（5′-3′）	Purpose
BD-ID16^{X}-F	catatggccatggaggccgaattcAACAAAGATATTCTGGATGTGCTTC	
BD-ID16^{X}-R	tagttatgcggccgctgcaggtcgacCTAGTTTCCATCTTCCAAAGAAAGG	
BD-ID17^{X}-F	catatggccatggaggccgaattcGTCAATGCATTCGTAAATATTATCTGG	
BD-ID17^{X}-R	tagttatgcggccgctgcaggtcgacTTATAACTTGCAGGATTGACTTGC	
BD-ID18^{X}-F	catatggccatggaggccgaattcGATACAGAGAGAGACATATTCCTTGA	
BD-ID18^{X}-R	tagttatgcggccgctgcaggtcgacTCAAGTTTGACCTAACATTCTTTCAC	
BD-ID19^{X}-F	catatggccatggaggccgaattcGATACAGAGAGAGAAATATTCCTTGAT	
BD-ID19^{X}-R	tagttatgcggccgctgcaggtcgacTCATTCAATTTTTCTTCTTTTCTGTTTTT	
BD-ID21^{X}-F	catatggccatggaggccgaattcCCCTTGGAAAAGGATATATTCCTTG	
BD-ID21^{X}-R	tagttatgcggccgctgcaggtcgacCTACTGCTCTTGAATCTTGCTCG	
BD-ID22^{X}-F	catatggccatggaggccgaattcGAGAAAAATATTTTCTTAGATATTGCATGT	
BD-ID22^{X}-R	tagttatgcggccgctgcaggtcgacTTAATCGAAGAATTTCATTTGTTGAATG	
BD-ID23^{X}-F	catatggccatggaggccgaattcGAAAAGAAGATACTTATGGATATTGCATG	
BD-ID23^{X}-R	tagttatgcggccgctgcaggtcgacTCACCCATTCCACACGGA	
BD-ID24^{X}-F	catatggccatggaggccgaattcGAAAAGAAGATATTTTTGGATATTGCATG	
BD-ID24^{X}-R	tagttatgcggccgctgcaggtcgacTTATTCTACTAACCTTCTAAAATAAATCCTAGA	
BD-ID25^{X}-F	catatggccatggaggccgaattcAAGGAAATCTTGAAGATACTTAAAGTAAGC	
BD-ID25^{X}-R	tagttatgcggccgctgcaggtcgacCTATTCCAAGTCGCGTATGGC	
BD-ID26^{X}-F	catatggccatggaggccgaattcGATGAAATCCAAGAGATTCTAAAAGTAAG	
BD-ID26^{X}-R	tagttatgcggccgctgcaggtcgacCTATGGTTTGTAGTCTGATGCAG	
BD-ID28^{X}-F	catatggccatggaggccgaattcAAAAAGATTCAAGACGTACTCAAAGT	
BD-ID28^{X}-R	tagttatgcggccgctgcaggtcgacCTAAAAAATAACCAAATGATGACTTATTTGG	
BD-ID29^{X}-F	catatggccatggaggccgaattcAAAGAGATCCAAGAGATACTTAAAGTG	
BD-ID29^{X}-R	tagttatgcggccgctgcaggtcgacTCATTGATTGTTGCGGCG	
BD-ID30^{X}-F	catatggccatggaggccgaattcAAAGAGATCCAAGAGATACTTAAAGTG	
BD-ID30^{X}-R	tagttatgcggccgctgcaggtcgacTTATGATTCTGAACTATTGAAATCATCAA	
BD-ID35^{X}-F	catatggccatggaggccgaattcGATAAAAGTATCTATATGATACTTAAAGTAAGCT	
BD-ID35^{X}-R	tagttatgcggccgctgcaggtcgacTTATGAACTAATGAGATCATCATTTAATTTTTT	
BD-ID36^{X}-F	catatggccatggaggccgaattcAAAAAGATCTATGACATACTTAAAGTAAGC	
BD-ID36^{X}-R	tagttatgcggccgctgcaggtcgacCTACAAATTGTGTCTCAACAACTTC	
BD-ID37^{X}-F	catatggccatggaggccgaattcGATACAGAGAAAGGAATATTCCTTGAT	
BD-ID37^{X}-R	tagttatgcggccgctgcaggtcgacTTAACCTTGATTTTTCTTATTTTCTGAG	
BD-ID38^{X}-F	catatggccatggaggccgaattcACTGAGCAGGAAATATTTCTAGACAT	
BD-ID38^{X}-R	tagttatgcggccgctgcaggtcgacCTAAATGTCAAAATATGCATAAGCTGG	
BD-ID39^{X}-F	catatggccatggaggccgaattcGATGATGAGAAAAACATTTTTCTTGAC	
BD-ID39^{X}-R	tagttatgcggccgctgcaggtcgacTTACCAGAAGTCACACATTACAAT	
qRT-PCR-SMV-F	CCGCTGAAACCCATTGTTGA	qRT-PCR for virus
qRT-PCR-SMV-R	GCGGGCCAGTTCTCTATCTC	titer detection
qRT-PCR-TMV-F	CACGACTGCCGAAACGTTAG	
qRT-PCR-TMV-R	TCAAGTTGCAGGACCAGAGG	
qRT-PCR-PPV-F	GAGAAGGCGAGGAGGAAGTT	
qRT-PCR-PPV-R	TGCAGGCTGTATGACTGGAG	

(continued table)

Primer name	Sequence (5′-3′)	Purpose
qRT-PCR-PCV-F	CAGTCTTACGAGCAGCGACA	
qRT-PCR-PCV-R	TGTCACGCACCAACCAAAAC	
qRT-PCR-BSMV-F	GAAAGTGTGCTGGCCTTTCG	
qRT-PCR-BSMV-R	CATCTGAGCGCTGACTCGTA	
qRT-PCR-TRV2-F	ACTACCAAGGCGAACACTGG	
qRT-PCR-TRV2-R	GAGGCGGTGCAGATGAACTA	
pCambia1300-ID1-F	gagaacacgggggacgagctcATGTCGAAGGCAGTTTCAGAATC	ORF sequence cloned in pCambia1300
pCambia1300-ID1-R	atttgttggatcccgggtaccTTATAAGATGGTGGGTTTATAAGGATC	
pCambia1300-ID2-F	gagaacacgggggacgagctcATGGCTTCTAAGGCCATCATCC	
pCambia1300-ID2-R	atttgttggatcccgggtaccCTACTGCTGCTTCCACTGT	
pCambia1300-ID3-F	gagaacacgggggacgagctcATGGAATTAGAGTCTTCTTC	
pCambia1300-ID3-R	atttgttggatcccgggtaccTTACAATTCTTCTCTGCGTGGAAATAC	
pCambia1300-ID5-F	gagaacacgggggacgagctcATGTTGTATTTTTTTGTCTA	
pCambia1300-ID5-R	atttgttggatcccgggtaccTTAAACATGGAGCGAGTCATG	
pCambia1300-ID7-F	gagaacacgggggacgagctcATGTCATCTTCTTCATTCTCAAC	
pCambia1300-ID7-R	atttgttggatcccgggtaccCTACTGCTCTTCATTTTTACTTGATA	
pCambia1300-ID8-F	gagaacacgggggacgagctcATGGAATGCAAGAAACAAGGAG	
pCambia1300-ID8-R	atttgttggatcccgggtaccTCAATCGTTGAACTTACTTAACACTG	
pCambia1300-ID9-F	gagaacacgggggacgagctcATGGCTGTGATGCTGTGTGGAG	
pCambia1300-ID9-R	atttgttggatcccgggtaccTTAATCAAACCCTTCATGCCTTAAG	
pCambia1300-ID10-F	gagaacacgggggacgagctcATGTCCAACAAAGCTGCTCC	
pCambia1300-ID10-R	atttgttggatcccgggtaccTCAGTCACTGTCATGCAATTC	
pCambia1300-ID11-F	gagaacacgggggacgagctcATGGCTTGGGGATCACG	
pCambia1300-ID11-R	atttgttggatcccgggtaccTTATATGTATTCACTAACAAAAGGTTGG	
pCambia1300-ID12-F	gagaacacgggggacgagctcATGGCTACGGGATCACCT	
pCambia1300-ID12-R	atttgttggatcccgggtaccTTACGTAACATGTAACATGTATCTCTC	
pCambia1300-ID14-F	gagaacacgggggacgagctcATGGCTTGGGGATCAAGTTC	
pCambia1300-ID14-R	atttgttggatcccgggtaccTTATCTGTATTCTGAAATATAAGGTTGGC	
pCambia1300-ID15-F	gagaacacgggggacgagctcATGACACAGCAATCATGTTCC	
pCambia1300-ID15-R	atttgttggatcccgggtaccTTACCTTAAAATCAATGGATTGACAG	
pCambia1300-ID16-F	gagaacacgggggacgagctcATGGCTTCCAACAACAACAG	
pCambia1300-ID16-R	atttgttggatcccgggtaccCTAGTTTCCATCTTCCAAAGAAAGG	
pCambia1300-ID17-F	gagaacacgggggacgagctcATGGCTTCCTCGGCCTT	
pCambia1300-ID17-R	atttgttggatcccgggtaccTTATAACTTGCAGGATTGACTTGC	
pCambia1300-ID18-F	gagaacacgggggacgagctcATGCCAACAGAGATTGCAATG	
pCambia1300-ID18-R	atttgttggatcccgggtaccTCAAGTTTGACCTAACATTCTTTCAC	
pCambia1300-ID19-F	gagaacacgggggacgagctcATGCCTTCCGATCGTGAAAT	
pCambia1300-ID19-R	atttgttggatcccgggtaccTCATTCAATTTTTCTTCTTTTCTGTTTTT	
pCambia1300-ID21-F	gagaacacgggggacgagctcATGGAATTCGTGGCTTCTTC	
pCambia1300-ID21-R	atttgttggatcccgggtaccCTACTGCTCTTGAATCTTGCTCG	
pCambia1300-ID23-F	gagaacacgggggacgagctcATGGCTTTCCAAGTTTCTCATA	
pCambia1300-ID23-R	atttgttggatcccgggtaccTCACCCATTCCACACGGA	

(continued table)

Primer name	Sequence（5′-3′）	Purpose
pCambia1300-ID24-F	gagaacacgggggacgagctcATGCCATATTCTTATTCGTTCCG	
pCambia1300-ID24-R	atttgttggatcccgggtaccTTATTCTACTAACCTTCTAAAATAAATCCTAGA	
pCambia1300-ID25-F	gagaacacgggggacgagctcATGAAGTTTGGTTCTATATTCCAACAG	
pCambia1300-ID25-R	atttgttggatcccgggtaccCTATTCCAAGTCGCGTATGGC	
pCambia1300-ID26-F	gagaacacgggggacgagctcATGGCTGCGACAACACG	
pCambia1300-ID26-R	atttgttggatcccgggtaccCTATGGTTTGTAGTCTGATGCAG	
pCambia1300-ID28-F	gagaacacgggggacgagctcATGTCCTCCTCCTCCATTAGCT	
pCambia1300-ID28-R	atttgttggatcccgggtaccCTAAAAAATAACCAAATGATGACTTATTTGG	
pCambia1300-ID29-F	gagaacacgggggacgagctcATGGCTAAGCAATCATCCTCAT	
pCambia1300-ID29-R	atttgttggatcccgggtaccTCATTGATTGTTGCGGCG	
pCambia1300-ID30-F	gagaacacgggggacgagctcATGGCTATGCAATCATCCTCA	
pCambia1300-ID30-R	atttgttggatcccgggtaccTTATGATTCTGAACTATTGAAATCATCAA	
pCambia1300-ID35-F	gagaacacgggggacgagctcATGGCTGTGCGATCATTCTC	
pCambia1300-ID35-R	atttgttggatcccgggtaccTTATGAACTAATGAGATCATCATTTAATTTTTT	
pCambia1300-ID36-F	gagaacacgggggacgagctcATGGCTCTGCGATCATTCT	
pCambia1300-ID36-R	atttgttggatcccgggtaccCTACAAATTGTGTCTCAACAACTTC	
pCambia1300-ID39-F	gagaacacgggggacgagctcATGCCCTCACTATTGCGAG	
pCambia1300-ID39-R	atttgttggatcccgggtaccTTACCAGAAGTCACACATTACAAT	
pC-SRC7^{TN}-F	gagaacacgggggacgagctcATGGCTGCAACGAAACGTTCCCTTGC	SRC7^{TN} cloned in
pC-SRC7^{TN}-R	TGTTTCCTGCTTTGTAGCATCA	pCambia1300
pC-SRC7^{TN}-ID1^{X}-F	tgatgctacaaagcaggaaacaTACCTCGAGAAGGAGGTTTTTC	ID^{X} sequence cloned
pC-SRC7^{TN}-ID1^{X}-R	atttgttggatcccgggtaccTTATAAGATGGTGGGTTTATAAGGATC	after SRC7^{TN} in
pC-SRC7^{TN}-ID2^{X}-F	tgatgctacaaagcaggaaacaAATGACAGGGAAATATTTTTAGATATTGC	pCambia1300
pC-SRC7^{TN}-ID2^{X}-R	atttgttggatcccgggtaccCTACTGCTGCTTCCACTGT	
pC-SRC7^{TN}-ID3^{X}-F	tgatgctacaaagcaggaaacaAAGGATATATTCCTTGATGTGTGT	
pC-SRC7^{TN}-ID3^{X}-R	atttgttggatcccgggtaccTTACAATTCTTCTCTGCGTGGAAATAC	
pC-SRC7^{TN}-ID4^{X}-F	tgatgctacaaagcaggaaacaGAAGATGAAAAGGGAATTTTCCTTG	
pC-SRC7^{TN}-ID4^{X}-R	atttgttggatcccgggtaccTTATACAACTTGATAATACAAGCAACC	
pC-SRC7^{TN}-ID5^{X}-F	tgatgctacaaagcaggaaacaCAGATTTTTCTAGATTTGGCATGTTT	
pC-SRC7^{TN}-ID5^{X}-R	atttgttggatcccgggtaccTTAAACATGGAGCGAGTCATG	
pC-SRC7^{TN}-ID7^{X}-F	tgatgctacaaagcaggaaacaGAAAAGGATATATTTCTTGATATATGTTGTT	
pC-SRC7^{TN}-ID7^{X}-R	atttgttggatcccgggtaccCTACTGCTCTTCATTTTTACTTGATA	
pC-SRC7^{TN}-ID8^{X}-F	tgatgctacaaagcaggaaacaTTTTCTGCAGATATAGGGACAAGAAG	
pC-SRC7^{TN}-ID8^{X}-R	atttgttggatcccgggtaccTCAATCGTTGAACTTACTTAACACTG	
pC-SRC7^{TN}-ID9^{X}-F	tgatgctacaaagcaggaaacaATTCCCAACAAAGAGGTTCAGAA	
pC-SRC7^{TN}-ID9^{X}-R	atttgttggatcccgggtaccTTAATCAAACCCTTCATGCCTTAAG	
pC-SRC7^{TN}-ID10^{X}-F	tgatgctacaaagcaggaaacaGATCGTAAAGAGCAACAAATTTTTCT	
pC-SRC7^{TN}-ID10^{X}-R	atttgttggatcccgggtaccTCAGTCACTGTCATGCAATTC	
pC-SRC7^{TN}-ID11^{X}-F	tgatgctacaaagcaggaaacaGAAGAAGAGAAGAAAGTTTTTCTTGAC	
pC-SRC7^{TN}-ID11^{X}-R	atttgttggatcccgggtaccTTATATGTATTCACTAACAAAAGGTTGG	

(continued table)

Primer name	Sequence (5′-3′)	Purpose
pC-SRC7^{TN}-ID12^{X}-F	tgatgctacaaagcaggaaacaATGGCTACGGGATCACCT	
pC-SRC7^{TN}-ID12^{X}-R	atttgttggatcccgggtaccTTACGTAACATGTAACATGTATCTCTC	
pC-SRC7^{TN}-ID13^{X}-F	tgatgctacaaagcaggaaacaGAAGAGAAAAGTGTTTTTCTTGACC	
pC-SRC7^{TN}-ID13^{X}-R	atttgttggatcccgggtaccTCAAACATCTACAAAAGGTGCAC	
pC-SRC7^{TN}-ID14^{X}-F	tgatgctacaaagcaggaaacaAAGGAAATTCTAGATGTACTTACAGTA	
pC-SRC7^{TN}-ID14^{X}-R	atttgttggatcccgggtaccTTATCTGTATTCTGAAATATAAGGTTGGC	
pC-SRC7^{TN}-ID15^{X}-F	tgatgctacaaagcaggaaacaAATGAGAAGGAAGTTTTCCTTGAC	
pC-SRC7^{TN}-ID15^{X}-R	atttgttggatcccgggtaccTTACCTTAAAATCAATGGATTGACAG	
pC-SRC7^{TN}-ID16^{X}-F	tgatgctacaaagcaggaaacaAACAAAGATATTCTGGATGTGCTTC	
pC-SRC7^{TN}-ID16^{X}-R	atttgttggatcccgggtaccCTAGTTTCCATCTTCCAAAGAAAGG	
pC-SRC7^{TN}-ID17^{X}-F	tgatgctacaaagcaggaaacaGTCAATGCATTCGTAAATATTATCTGG	
pC-SRC7^{TN}-ID17^{X}-R	atttgttggatcccgggtaccTTATAACTTGCAGGATTGACTTGC	
pC-SRC7^{TN}-ID18^{X}-F	tgatgctacaaagcaggaaacaGATACAGAGAGAGACATATTCCTTGA	
pC-SRC7^{TN}-ID18^{X}-R	atttgttggatcccgggtaccTCAAGTTTGACCTAACATTCTTTCAC	
pC-SRC7^{TN}-ID19^{X}-F	tgatgctacaaagcaggaaacaGATACAGAGAGAGAAATATTCCTTGAT	
pC-SRC7^{TN}-ID19^{X}-R	atttgttggatcccgggtaccTCATTCAATTTTTCTTCTTTTCTGTTTTT	
pC-SRC7^{TN}-ID21^{X}-F	tgatgctacaaagcaggaaacaCCCTTGGAAAAGGATATATTCCTTG	
pC-SRC7^{TN}-ID21^{X}-R	atttgttggatcccgggtaccCTACTGCTCTTGAATCTTGCTCG	
pC-SRC7^{TN}-ID22^{X}-F	tgatgctacaaagcaggaaacaGAGAAAAATATTTTCTTAGATATTGCATGT	
pC-SRC7^{TN}-ID22^{X}-R	atttgttggatcccgggtaccTTAATCGAAGAATTTCATTTGTTGAATG	
pC-SRC7^{TN}-ID23^{X}-F	tgatgctacaaagcaggaaacaGAAAAGAAGATACTTATGGATATTGCATG	
pC-SRC7^{TN}-ID23^{X}-R	atttgttggatcccgggtaccTCACCCATTCCACACGGA	
pC-SRC7^{TN}-ID24^{X}-F	tgatgctacaaagcaggaaacaGAAAAGAAGATATTTTTGGATATTGCATG	
pC-SRC7^{TN}-ID24^{X}-R	atttgttggatcccgggtaccTTATTCTACTAACCTTCTAAAATAAATCCTAGA	
pC-SRC7^{TN}-ID25^{X}-F	tgatgctacaaagcaggaaacaAAGGAAATCTTGAAGATACTTAAAGTAAGC	
pC-SRC7^{TN}-ID25^{X}-R	atttgttggatcccgggtaccCTATTCCAAGTCGCGTATGGC	
pC-SRC7^{TN}-ID26^{X}-F	tgatgctacaaagcaggaaacaGATGAAATCCAAGAGATTCTAAAAGTAAG	
pC-SRC7^{TN}-ID26^{X}-R	atttgttggatcccgggtaccCTATGGTTTGTAGTCTGATGCAG	
pC-SRC7^{TN}-ID28^{X}-F	tgatgctacaaagcaggaaacaAAAAAGATTCAAGACGTACTCAAAGT	
pC-SRC7^{TN}-ID28^{X}-R	atttgttggatcccgggtaccCTAAAAAATAACCAAATGATGACTTATTTGG	
pC-SRC7^{TN}-ID29^{X}-F	tgatgctacaaagcaggaaacaAAAGAGATCCAAGAGATACTTAAAGTG	
pC-SRC7^{TN}-ID29^{X}-R	atttgttggatcccgggtaccTCATTGATTGTTGCGGCG	
pC-SRC7^{TN}-ID30^{X}-F	tgatgctacaaagcaggaaacaAAAGAGATCCAAGAGATACTTAAAGTG	
pC-SRC7^{TN}-ID30^{X}-R	atttgttggatcccgggtaccTTATGATTCTGAACTATTGAAATCATCAA	
pC-SRC7^{TN}-ID35^{X}-F	tgatgctacaaagcaggaaacaGATAAAAGTATCTATATGATACTTAAAGTAAGCT	
pC-SRC7^{TN}-ID35^{X}-R	atttgttggatcccgggtaccTTATGAACTAATGAGATCATCATTTAATTTTTT	
pC-SRC7^{TN}-ID36^{X}-F	tgatgctacaaagcaggaaacaAAAAAGATCTATGACATACTTAAAGTAAGC	
pC-SRC7^{TN}-ID36^{X}-R	atttgttggatcccgggtaccCTACAAATTGTGTCTCAACAACTTC	
pC-SRC7^{TN}-ID37^{X}-F	tgatgctacaaagcaggaaacaGATACAGAGAAAGGAATATTCCTTGAT	
pC-SRC7^{TN}-ID37^{X}-R	atttgttggatcccgggtaccTTAACCTTGATTTTTCTTATTTTCTGAG	
pC-SRC7^{TN}-ID38^{X}-F	tgatgctacaaagcaggaaacaACTGAGCAGGAAATATTTCTAGACAT	
pC-SRC7^{TN}-ID38^{X}-R	atttgttggatcccgggtaccCTAAATGTCAAAATATGCATAAGCTGG	

(continued table)

Primer name	Sequence（5′-3′）	Purpose
pC-SRC7^{TN}-ID39^{X}-F	tgatgctacaaagcaggaaacaGATGATGAGAAAAACATTTTTCTTGAC	
pC-SRC7^{TN}-ID39^{X}-R	atttgttggatcccgggtaccTTACCAGAAGTCACACATTACAAT	
BD-SRZ4-F	catatggccatggaggccgaattcATGGCTTGGGGATCAAGTTC	
BD-SRZ4-R	tagttatgcggccgctgcaggtcgacTTATCTGTATTCTGAAATATAAGGTTGGC	
pCambia1300-SRZ4^{TIR}-F	gagaacacgggggacgagctcATGGCTTGGGGATCAAGTTC	SRZ4 truncated
pCambia1300-SRZ4^{TIR}-R	atttgttggatcccgggtaccTTATGGGTAATCCGCAACATGTA	sequence cloned in
pCambia1300-SRZ4^{NBS}-F	gagaacacgggggacgagctcATGGACTAGAGTCACGAGTGCTAGA	pCambia1300
pCambia1300-SRZ4^{NBS}-R	atttgttggatcccgggtaccTTACTCCAAAGCATCAAAGCTTAC	
pCambia1300-SRZ4^{TN}-F	gagaacacgggggacgagctcATGGCTTGGGGATCAAGTTC	
pCambia1300-SRZ4^{TN}-R	atttgttggatcccgggtaccTTACTCCAAAGCATCAAAGCTTAC	
pCambia1300-SRZ4^{TNL}-F	gagaacacgggggacgagctcATGGCTTGGGGATCAAGTTC	
pCambia1300-SRZ4^{TNL}-R	atttgttggatcccgggtaccTTAACGCAAGCAGCCACTC	
pCambia1300-SRZ4^{TNLZ}-F	gagaacacgggggacgagctcATGGCTTGGGGATCAAGTTC	
pCambia1300-SRZ4^{TNLZ}-R	gagaacacgggggacgagctcTTAACTTCTCCAAAATGCAGCCA	
pCambia1300-SRZ4^{NLZ3}-F	gagaacacgggggacgagctcATGGACTAGAGTCACGAGTGCTAGA	
pCambia1300-SRZ4^{NLZ3}-R	gagaacacgggggacgagctcTTATAAACCGAATTTGGCAA	
pCambia1300-SRZ4^{Z3}-F	gagaacacgggggacgagctcATGAATGTGAGTGGTGCCTATG	
pCambia1300-SRZ4^{Z3}-R	gagaacacgggggacgagctcTTATAAACCGAATTTGGCAA	
pQBV3-ID3^{X}-F	AAAGCAGGCTCAGGGgatatcATGAAGGATATATTCCTTGATGTGTGT	ID^{X} sequence cloned
pQBV3-ID3^{X}-R	AGCTGGGTGCAGGGCgatatcCAATTCTTCTCTGCGTGGAAATAC	in entry vector
pQBV3-ID13^{X}-F	AAAGCAGGCTCAGGGgatatcATGGAAGAGAAAAGTGTTTTTCTTGACC	pQBV3
pQBV3-ID13^{X}-R	AGCTGGGTGCAGGGCgatatcAACATCTACAAAAGGTGCAC	
pQBV3-ID16^{X}-F	AAAGCAGGCTCAGGGgatatcATGAACAAAGATATTCTGGATGTGCTTC	
pQBV3-ID16^{X}-R	AGCTGGGTGCAGGGCgatatcGTTTCCATCTTCCAAAGAAAGG	
pQBV3-ID25^{X}-F	AAAGCAGGCTCAGGGgatatcATGAAGGAAATCTTGAAGATACTTAAAG	
pQBV3-ID25^{X}-R	AGCTGGGTGCAGGGCgatatcTTCCAAGTCGCGTATGGC	
pQBV3-ID29^{X}-F	AAAGCAGGCTCAGGGgatatcATGAAAGAGATCCAAGAGATACTTAAAG	
pQBV3-ID29^{X}-R	AGCTGGGTGCAGGGCgatatcTTGATTGTTGCGGCG	
pQBV3-ID30^{X}-F	AAAGCAGGCTCAGGGgatatcATGAAAGAGATCCAAGAGATACTTAAAG	
pQBV3-ID30^{X}-R	AGCTGGGTGCAGGGCgatatcTGATTCTGAACTATTGAAATCATCAA	
pQBV3-ID35^{X}-F	AAAGCAGGCTCAGGGgatatcATGGATAAAAGTATCTATATGATACTTA	
pQBV3-ID35^{X}-R	AGCTGGGTGCAGGGCgatatcTGAACTAATGAGATCATCATTTAATTTTT	
pQBV3-CP-F	AAAGCAGGCTCAGGGgatatcATGTCAGGCAAGGAGAAAGAAGG	
pQBV3-CP-R	AGCTGGGTGCAGGGCgatatcCTGCTGTGGGCCCATGCCCAG	
pQBV3-P3-F	AAAGCAGGCTCAGGGgatatcATGGGTGATGCGCAACAAAGGATG	
pQBV3-P3-R	AGCTGGGTGCAGGGCgatatcCTGTGCGGAAACATCTTCTG	
pQBV3-6K2-F	AAAGCAGGCTCAGGGgatatcATGAGCAAACATGAGGTGAGCA	
pQBV3-6K2-R	AGCTGGGTGCAGGGCgatatcTTGAGTTGATACTGGTTCACG	
pQBV3-P1-F	AAAGCAGGCTCAGGGgatatcATGGCAACAATCATGATCGGAAG	
pQBV3-P1-R	AGCTGGGTGCAGGGCgatatcGTAATGTTGAATATCCTCTATG	
pQBV3-NIb-F	AAAGCAGGCTCAGGGgatatcATGGGGAAAAAGGAAAGATGGGTT	
pQBV3-NIb-R	AGCTGGGTGCAGGGCgatatcTTGCAAAGAAACTGATTCAC	

(continued table)

Primer name	Sequence（5′-3′）	Purpose
pQBV3-HC-Pro-F	AAAGCAGGCTCAGGGgatatcATGTCCCAAACTCCTGAAGCTC	
pQBV3-HC-Pro-R	AGCTGGGTGCAGGGCgatatcACCAACTCTATAAAATTTCATC	
pQBV3-SRZ4-F	AAAGCAGGCTCAGGGgatatcATGGCTTGGGGATCAAGTTC	
pQBV3-SRZ4-R	AGCTGGGTGCAGGGCgatatcTCTGTATTCTGAAATATAAGGTTGGC	
T7	TAATACGACTCACTATAGG	AD universal primers for screening
3′AD	AGATGGTGCACGATGCACAG	candidate proteins

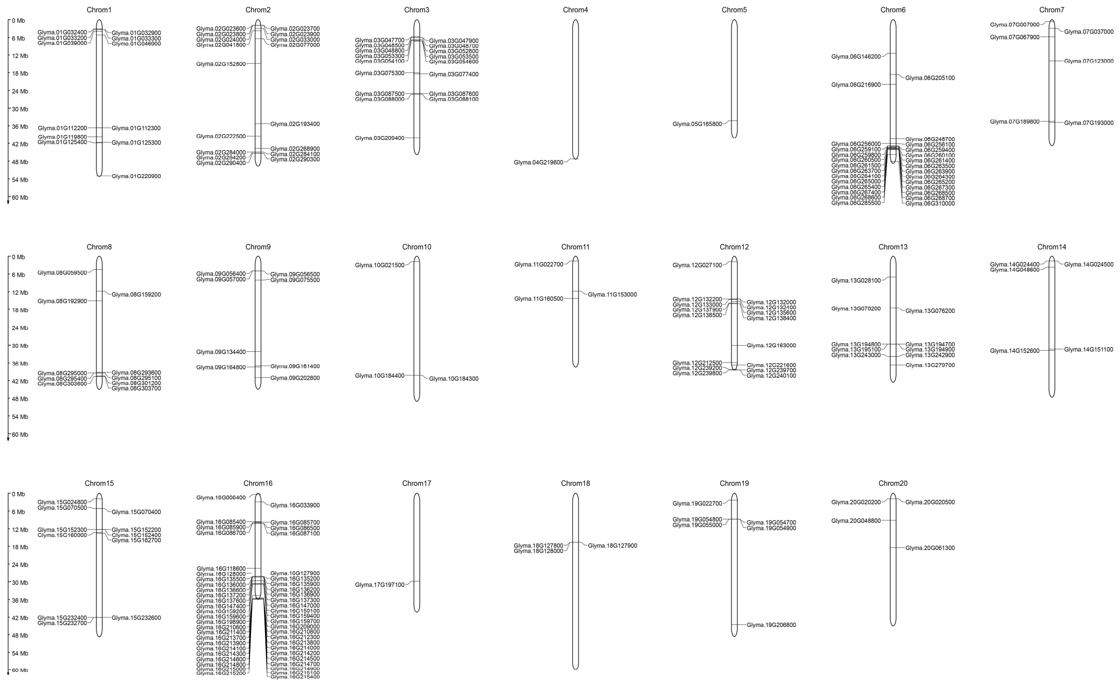

Figure S1. Chromosomal distribution of 210 *TNL-like* genes in soybean. The soybean genome consists of 20 pairs of chromosomes, and these 210 *TNL-like* genes are distributed on all 20 pairs of chromosomes in soybean. The left y-axis represents chromosome length, a cylindrical shape represents a chromosome, and the short lines connected to it represent the coding position of the gene. This image was drawn by the Mapgene2chrom website (http://mg2c.iask.in/mg2c_v2.0/) (accessed on 18 December 2020).

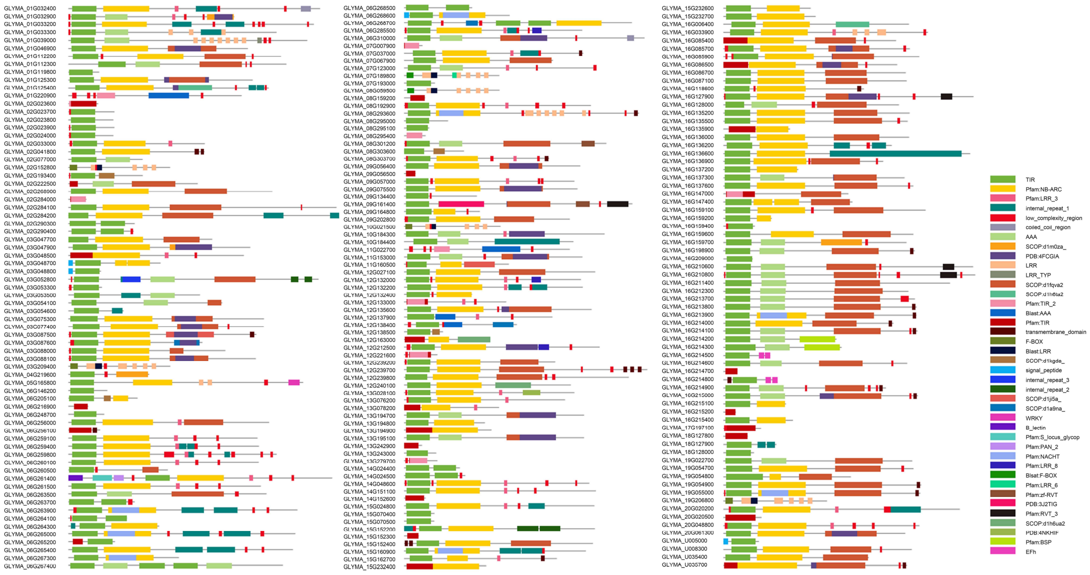

Figure S2. Structural domain prediction analysis of 210 TNL-like proteins in soybean. FASTA format corresponding to candidate genes was obtained from the NCBI database through gene number, and BATCH SMART in TBtools was used for domain predictions.

GLYMA_01G032400

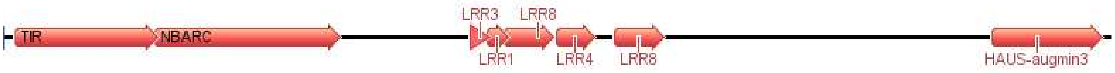

GLYMA_01G032900

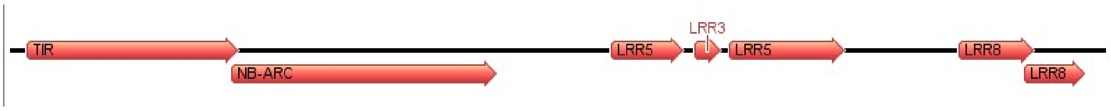

GLYMA_01G033200

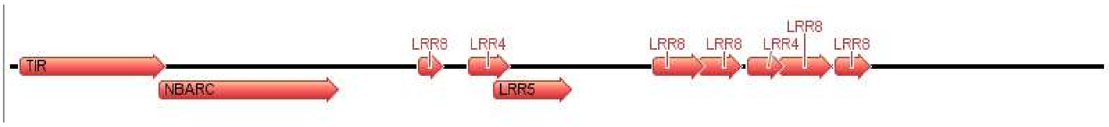

GLYMA_01G033300

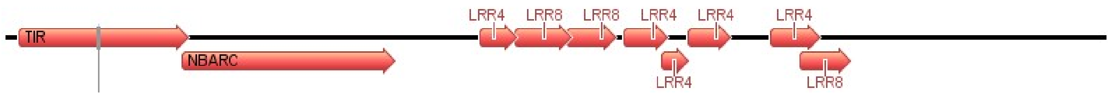

GLYMA_01G039000

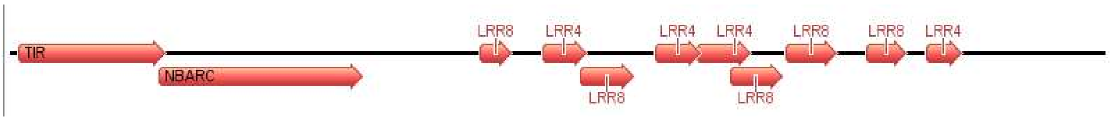

GLYMA_01G046900

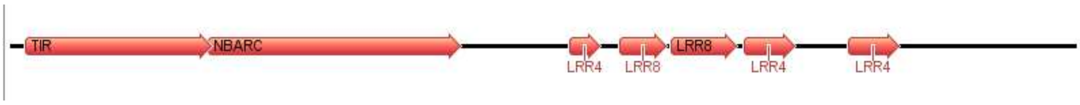

GLYMA_01G112200

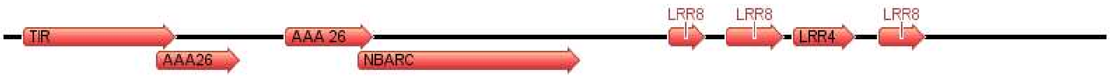

GLYMA_01G112300

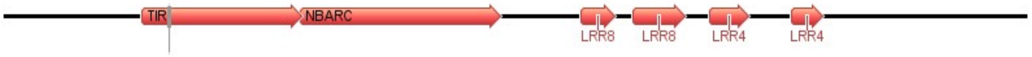

GLYMA_01G119800

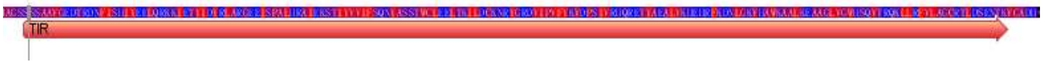

GLYMA_01G125300

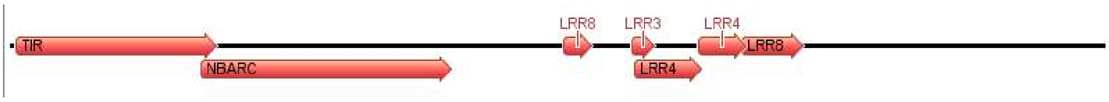

GLYMA_01G125400

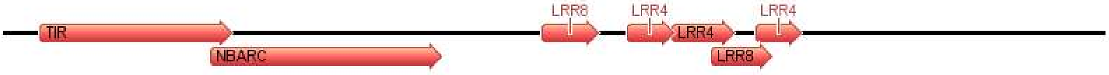

GLYMA_01G220900

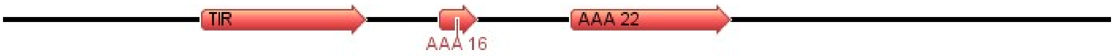

GLYMA_02G023600

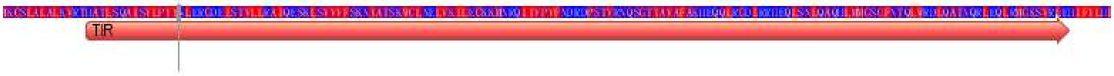

GLYMA_02G023700

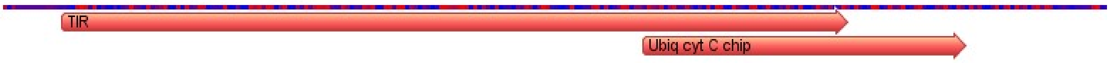

GLYMA_02G023800

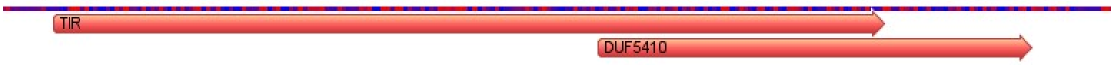

GLYMA_02G023900

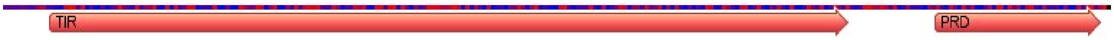

GLYMA_02G024000

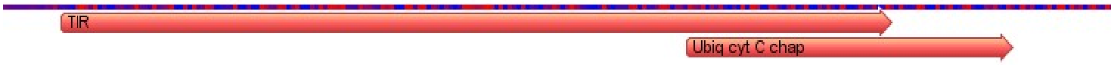

GLYMA_02G033000

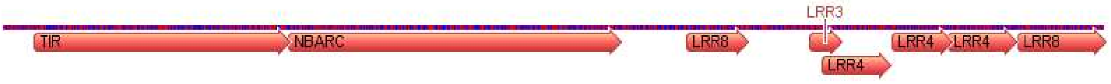

GLYMA_02G041800

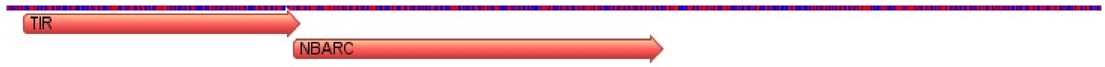

GLYMA_02G077000

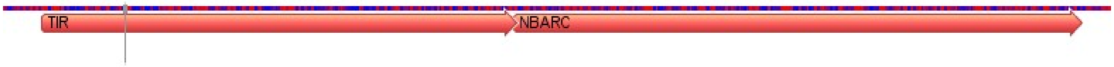

GLYMA_02G193400

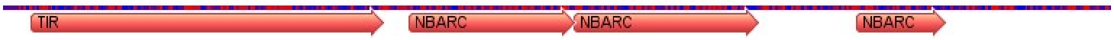

GLYMA_02G222500

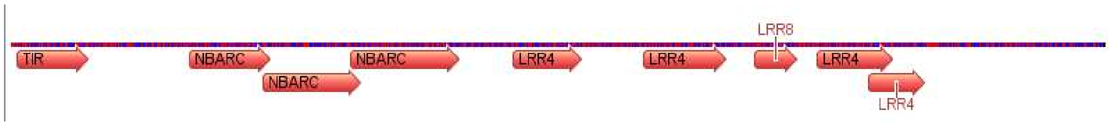

GLYMA_02G268900

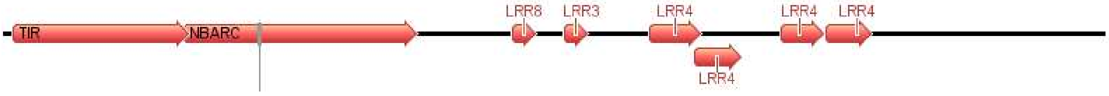

GLYMA_02G284000

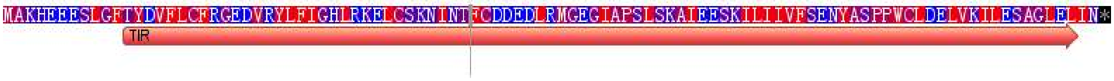

GLYMA_02G284100

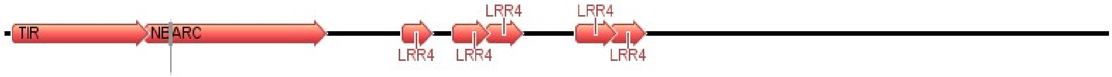

GLYMA_02G284200

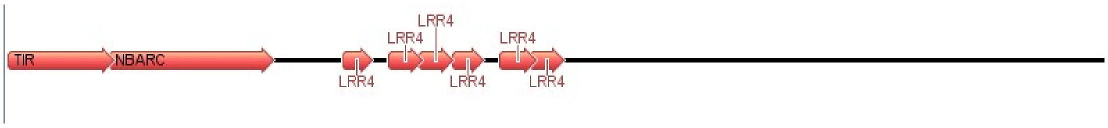

GLYMA_02G290300

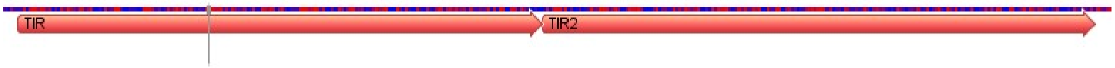

GLYMA_02G290400

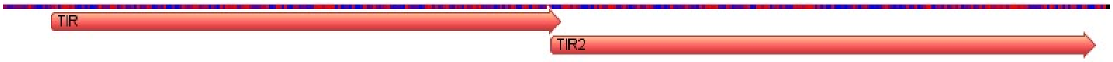

GLYMA_03G047700

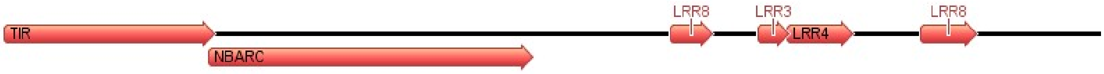

GLYMA_03G047900

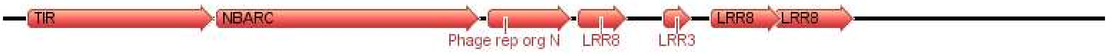

GLYMA_03G048500

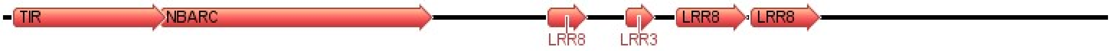

GLYMA_03G048700

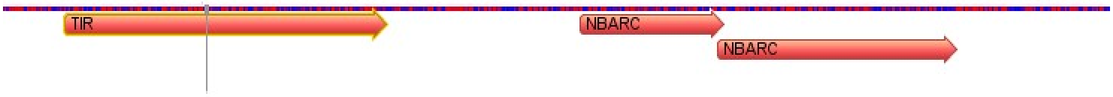

GLYMA_03G048800

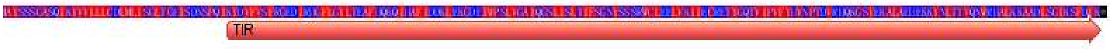

GLYMA_03G052800

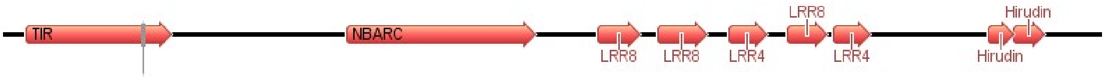

GLYMA_03G053300

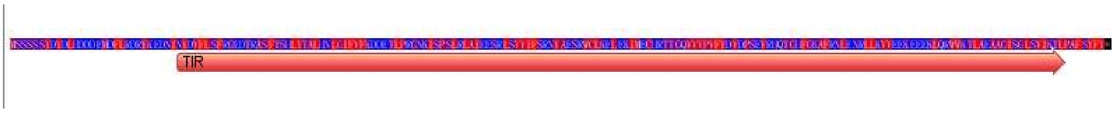

GLYMA_03G053500

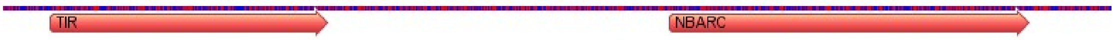

GLYMA_03G054100

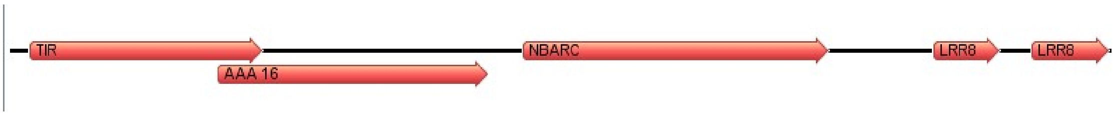

GLYMA_03G054600

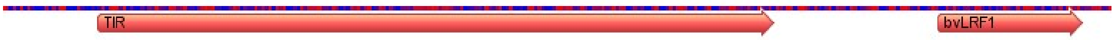

GLYMA_03G075300

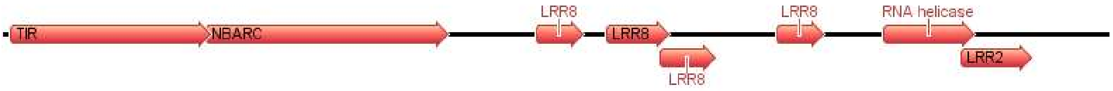

GLYMA_03G077400

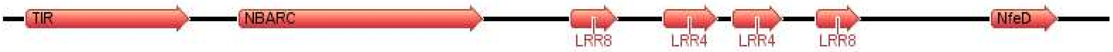

GLYMA_03G087500

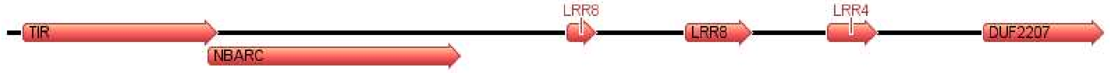

GLYMA_03G087600

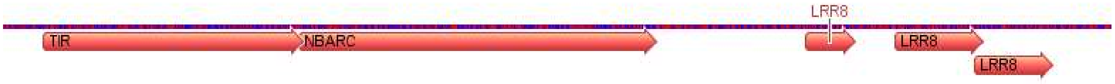

GLYMA_03G088000

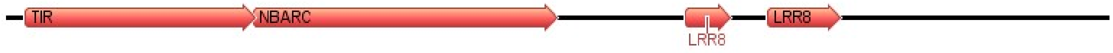

GLYMA_03G088100

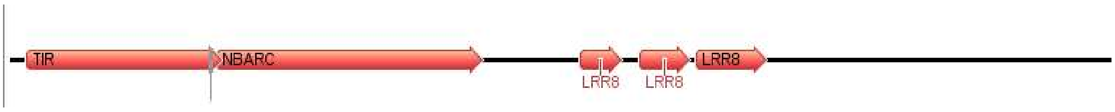

GLYMA_04G219600

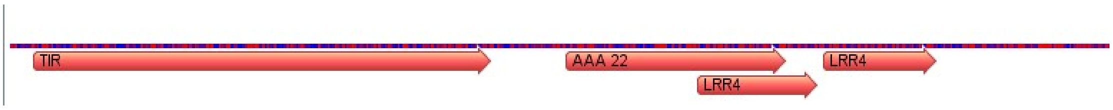

GLYMA_05G165800

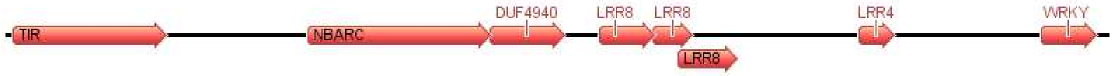

GLYMA_06G146200

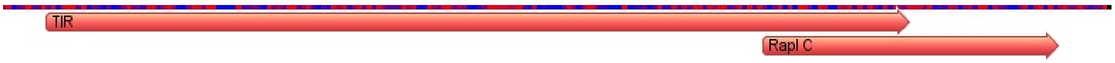

GLYMA_06G205100

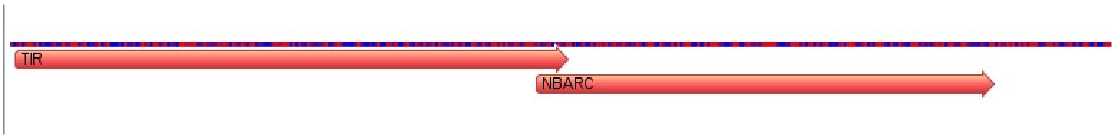

GLYMA_06G2016900

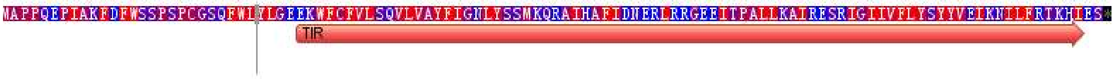

GLYMA_06G248700

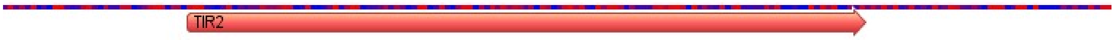

GLYMA_06G256000

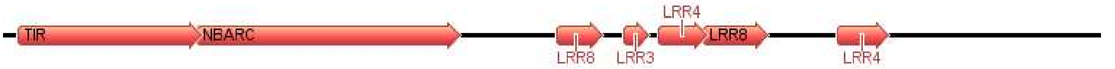

GLYMA_06G256100

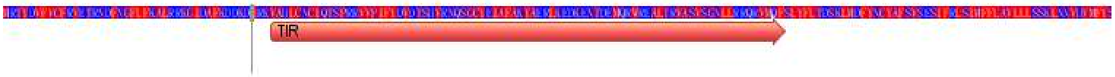

GLYMA_06G259100

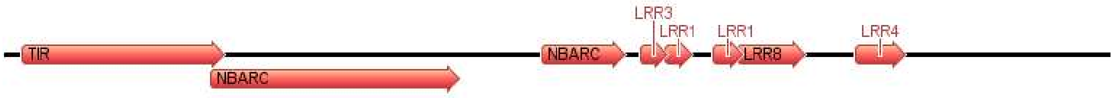

GLYMA_06G259400

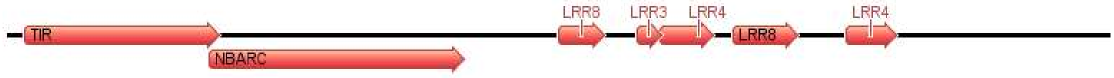

GLYMA_06G259800

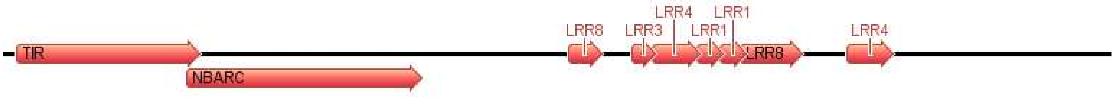

GLYMA_06G260100

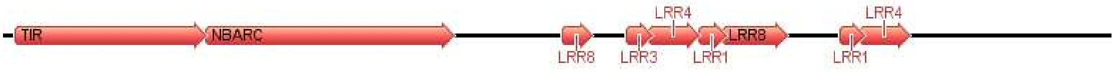

GLYMA_06G260500

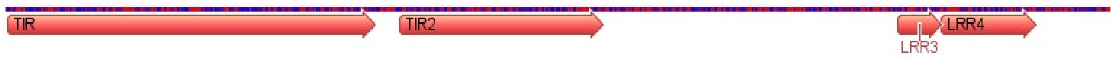

GLYMA_06G261400

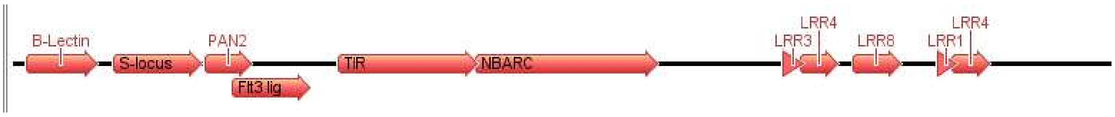

GLYMA_06G261500

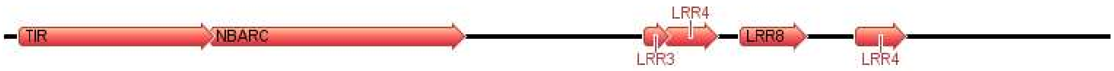

GLYMA_06G263500

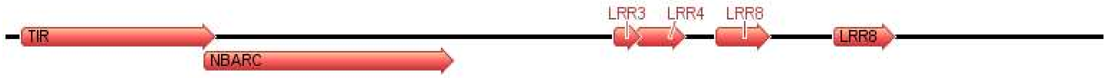

GLYMA_06G263700

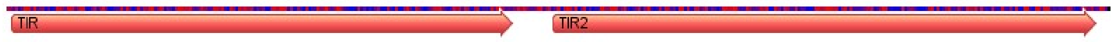

GLYMA_06G263900

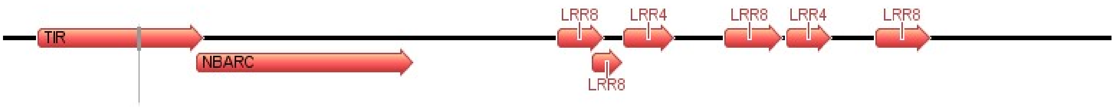

GLYMA_06G264100

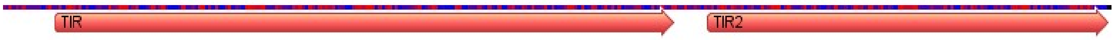

GLYMA_06G264300

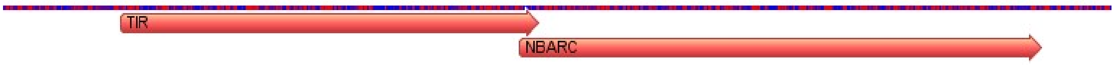

GLYMA_06G265000

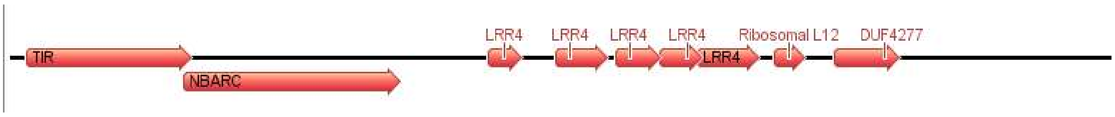

GLYMA_06G265200

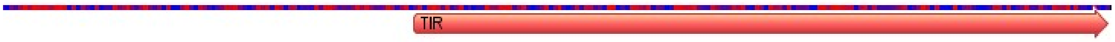

GLYMA_06G265400

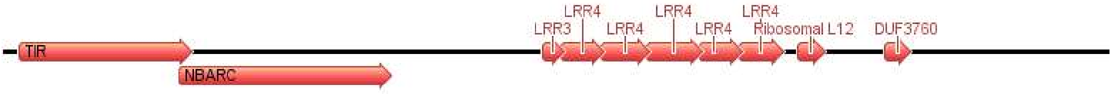

GLYMA_06G267300

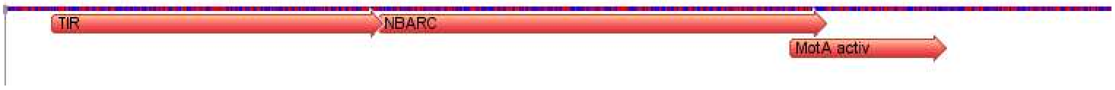

GLYMA_06G267400

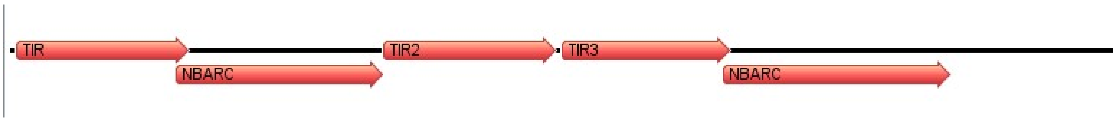

GLYMA_06G268500

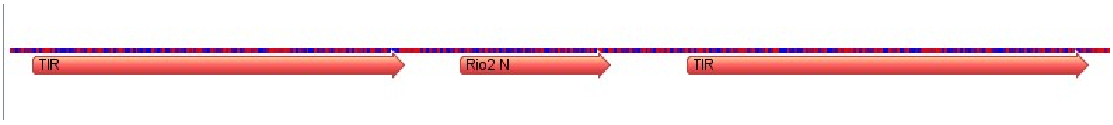

GLYMA_06G268600

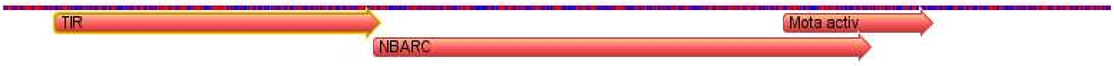

GLYMA_06G268700

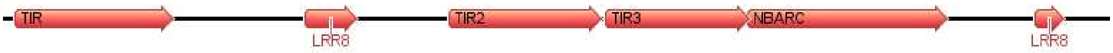

GLYMA_06G285500

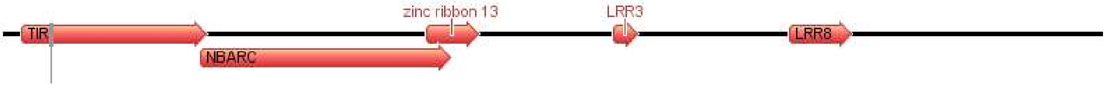

GLYMA_06G310000

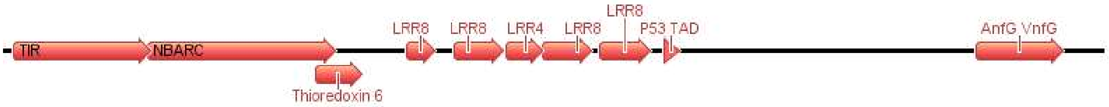

GLYMA_07G007900

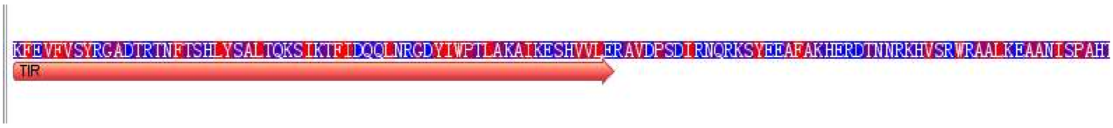

GLYMA_07G037000

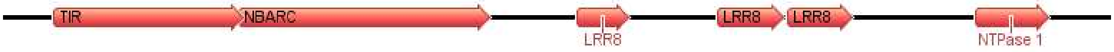

GLYMA_07G067900

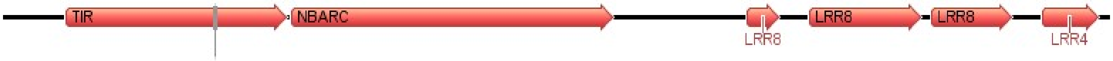

GLYMA_07G123000

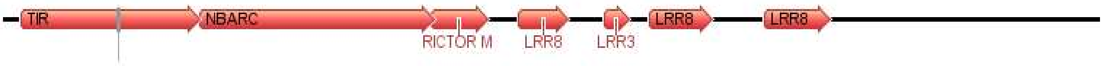

GLYMA_07G193000

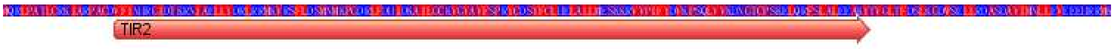

GLYMA_08G159200

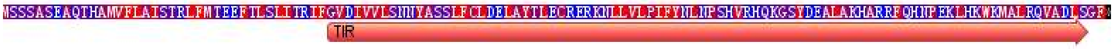

GLYMA_08G192900

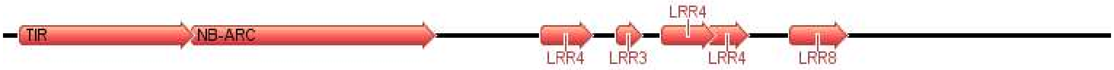

GLYMA_08G293600

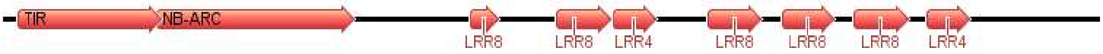

GLYMA_08G295000

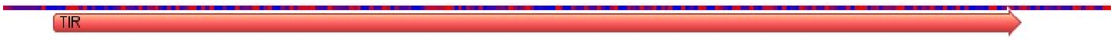

GLYMA_08G295100

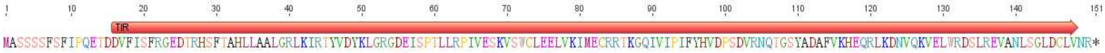

GLYMA_08G295400

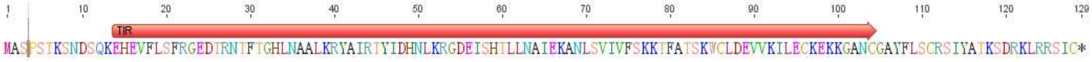

GLYMA_08G301200

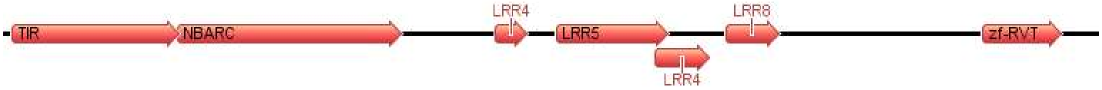

GLYMA_08G303600

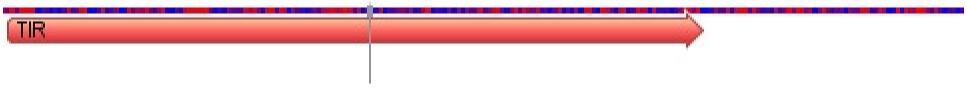

GLYMA_08G303700

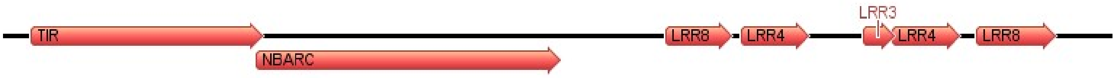

GLYMA_09G056400

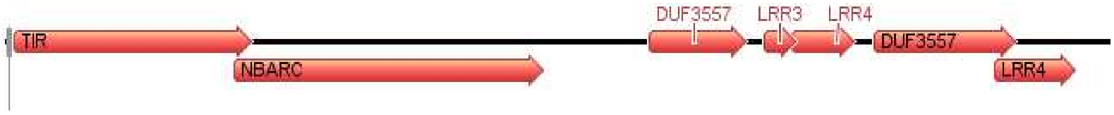

GLYMA_09G056500

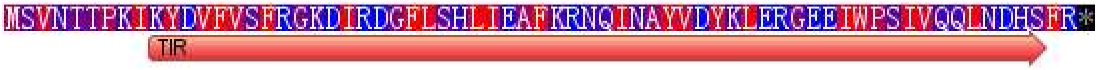

GLYMA_09G057000

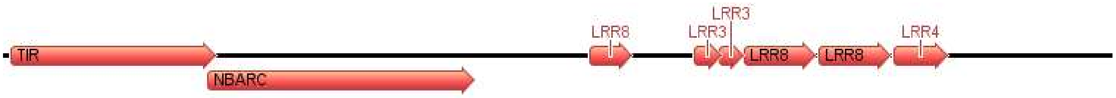

GLYMA_09G075500

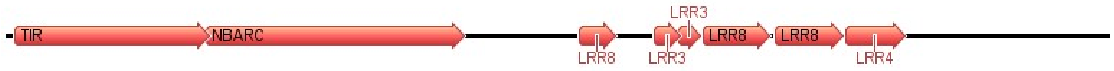

GLYMA_09G134400

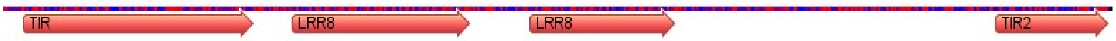

GLYMA_09G161400

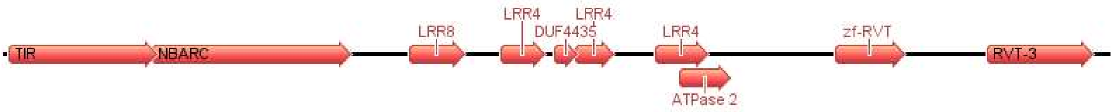

GLYMA_09G164800

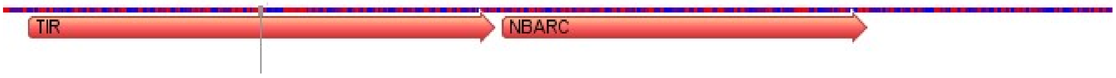

GLYMA_09G202800

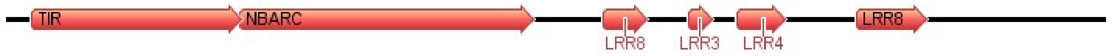

GLYMA_10G184300

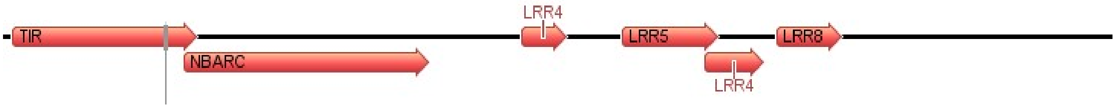

GLYMA_10G184400

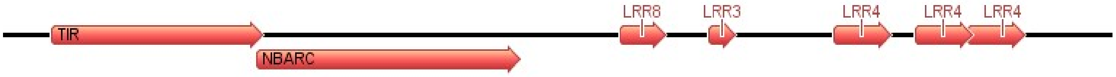

GLYMA_11G022700

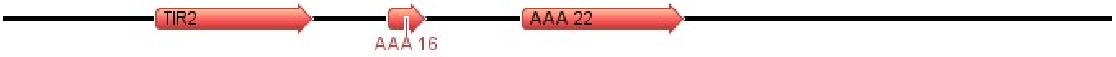

GLYMA_11G153000

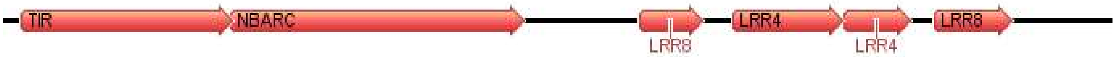

GLYMA_11G160500

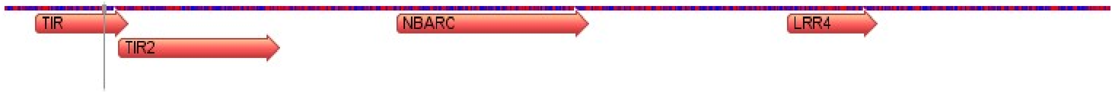

GLYMA_12G027100

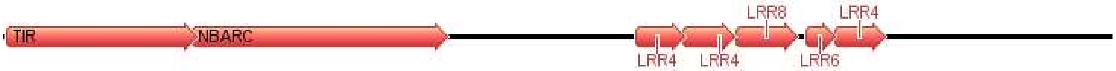

GLYMA_12G132000

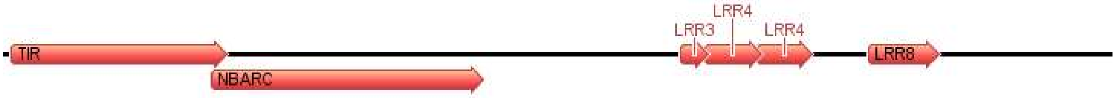

GLYMA_12G132200

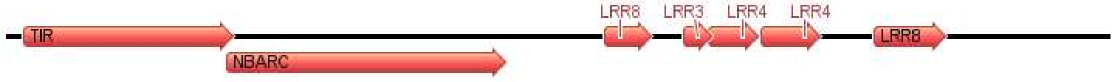

GLYMA_12G132400

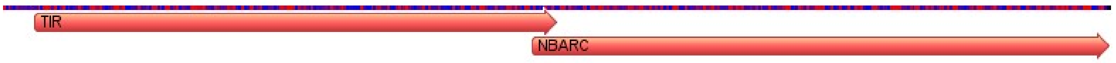

GLYMA_12G133000

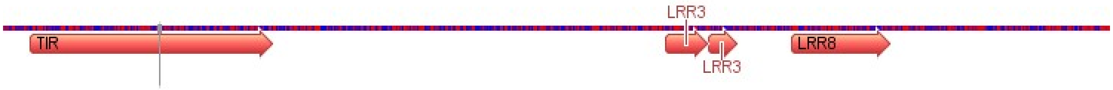

GLYMA_12G135600

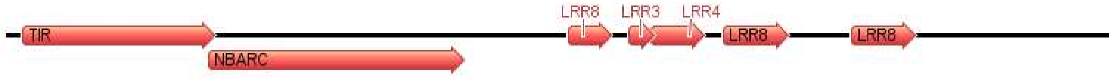

GLYMA_12G137900

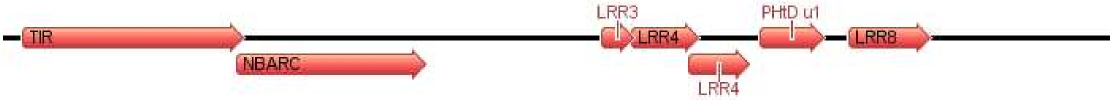

GLYMA_12G138400

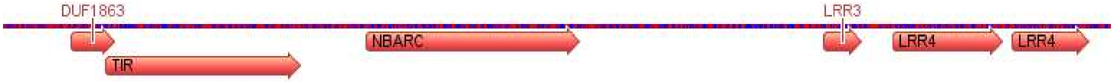

GLYMA_12G138500

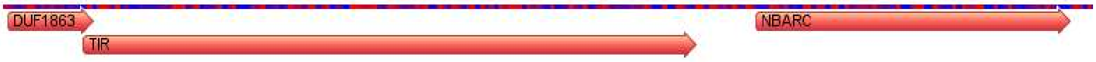

GLYMA_12G163000

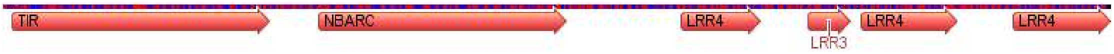

GLYMA_12G212500

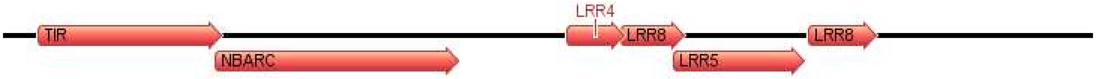

GLYMA_12G221600

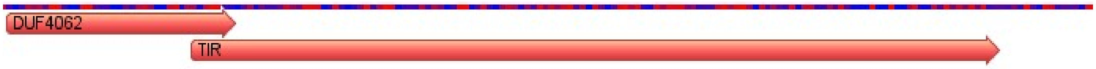

GLYMA_12G239200

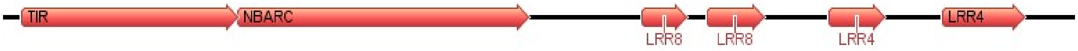

GLYMA_12G239700

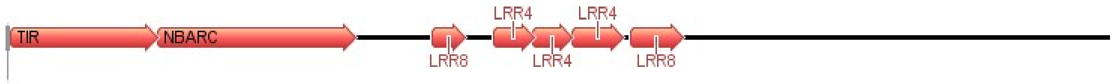

GLYMA_12G239800

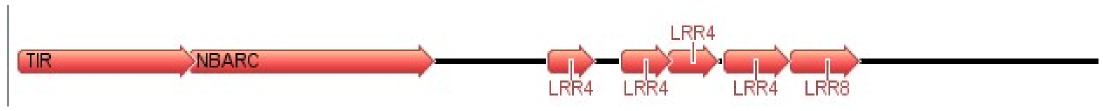

GLYMA_12G240100

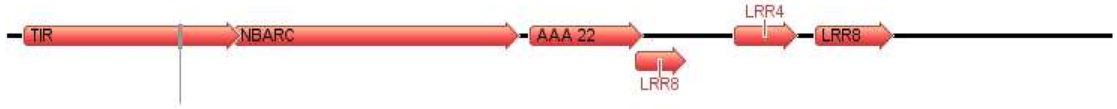

GLYMA_13G028100

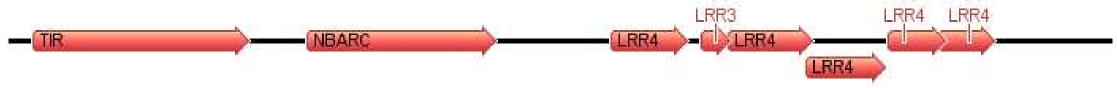

GLYMA_13G076200

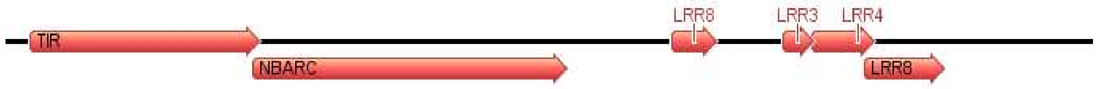

GLYMA_13G078200

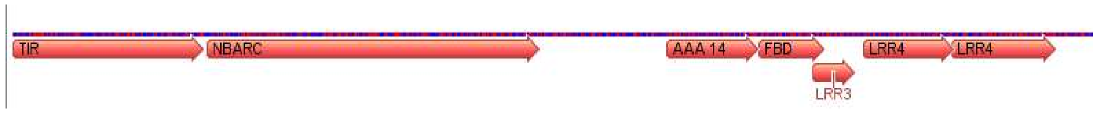

GLYMA_13G194700

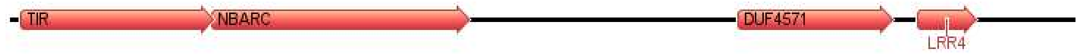

GLYMA_13G194800

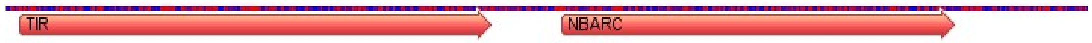

GLYMA_13G194900

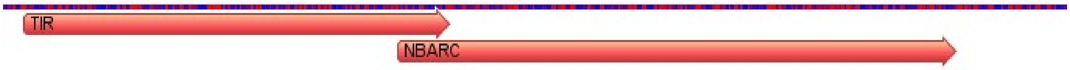

GLYMA_13G195100

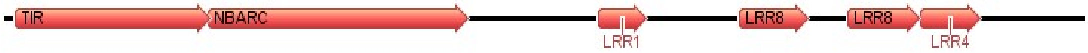

GLYMA_13G242900

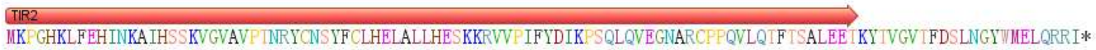

GLYMA_13G243000

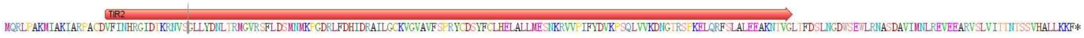

GLYMA_13G279700

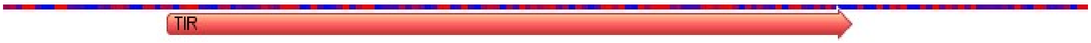

GLYMA_14G024400

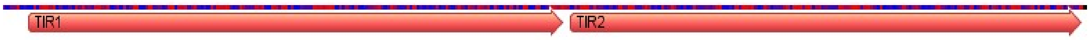

GLYMA_14G024500

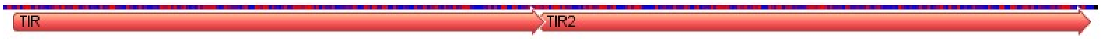

GLYMA_14G048600

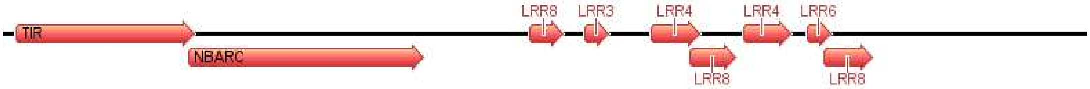

GLYMA_14G151100

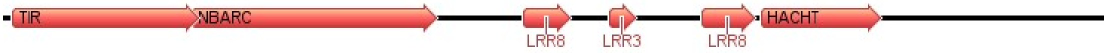

GLYMA_14G152600

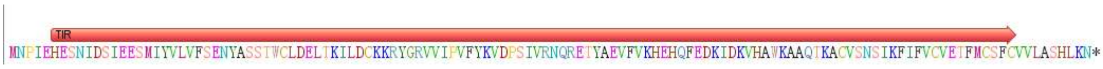

GLYMA_15G024800

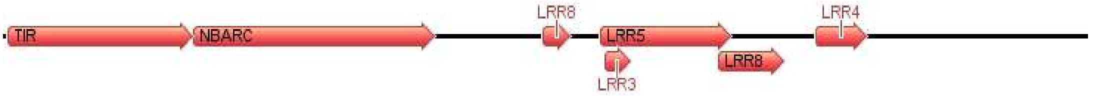

GLYMA_15G070400

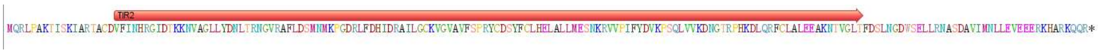

GLYMA_15G070500

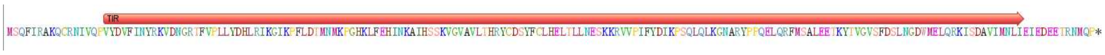

GLYMA_15G152200

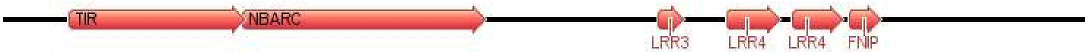

GLYMA_15G152300

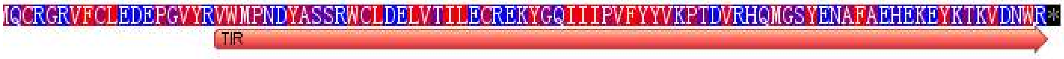

GLYMA_15G152400

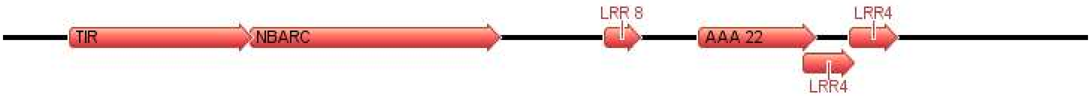

GLYMA_15G160900

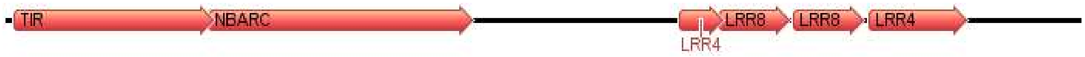

GLYMA_15G162700

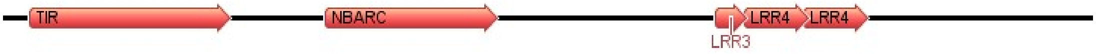

GLYMA_15G232400

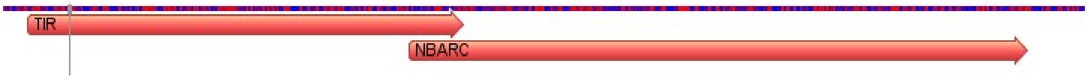

GLYMA_15G232600

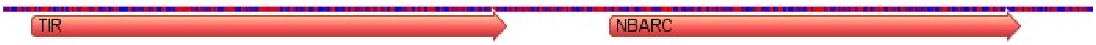

GLYMA_15G232700

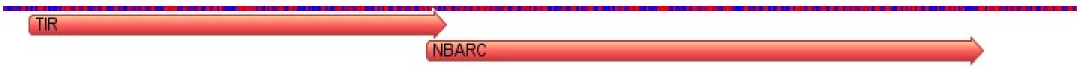

GLYMA_16G006400

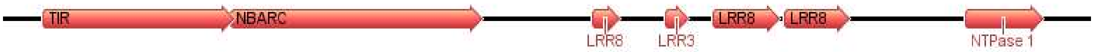

GLYMA_16G033900

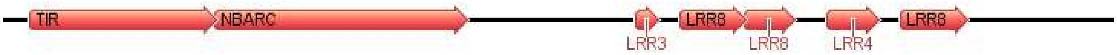

GLYMA_16G085400

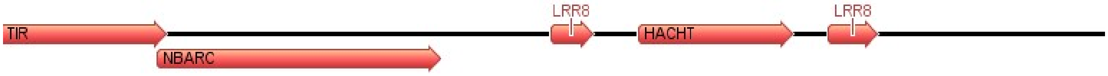

GLYMA_16G085700

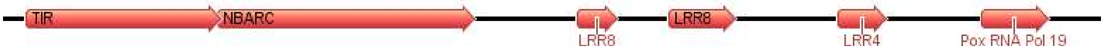

GLYMA_16G085900

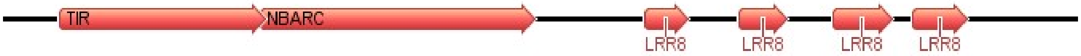

GLYMA_16G086500

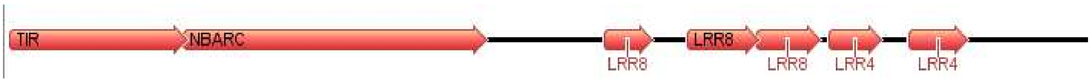

GLYMA_16G086700

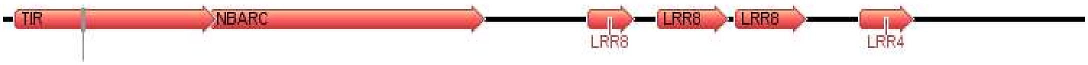

GLYMA_16G087100

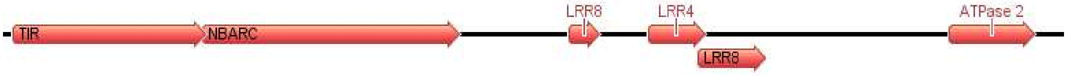

GLYMA_16G118600

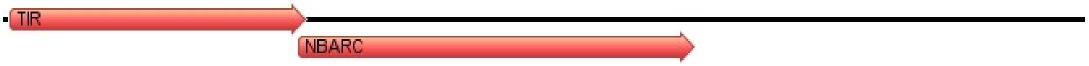

GLYMA_16G127900

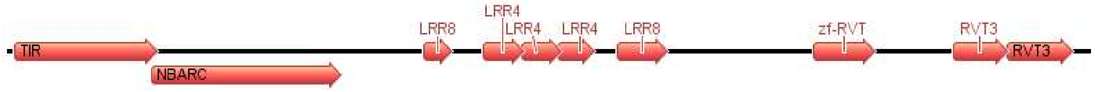

GLYMA_16G128000

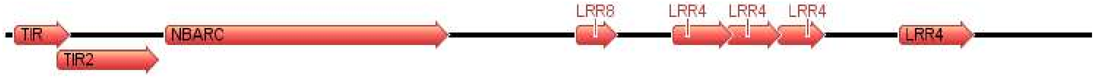

GLYMA_16G135200

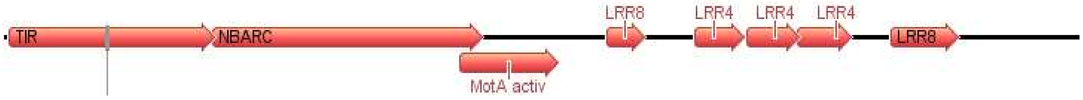

GLYMA_16G135500

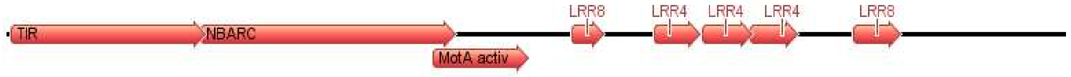

GLYMA_16G135900

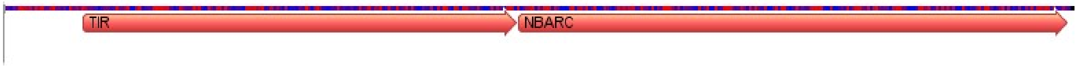

GLYMA_16G136000

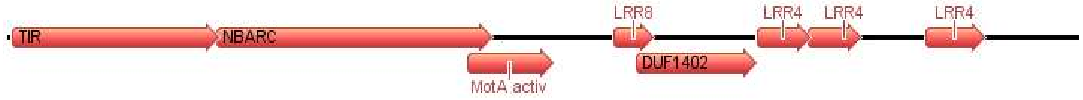

GLYMA_16G136200

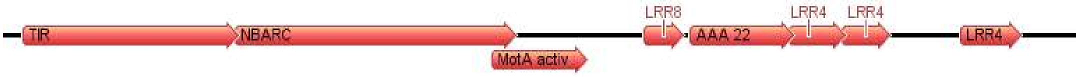

GLYMA_16G136600

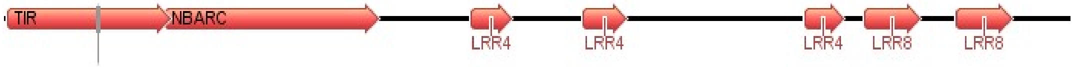

GLYMA_16G136900

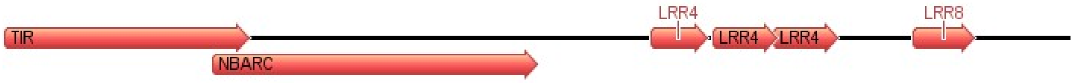

GLYMA_16G137200

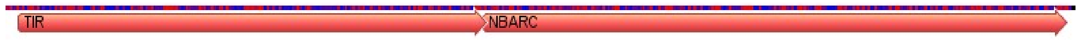

GLYMA_16G137300

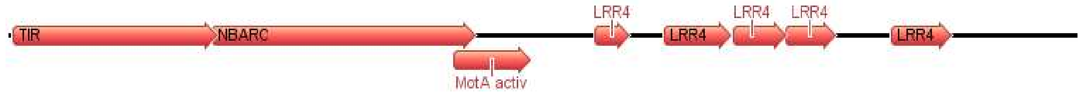

GLYMA_16G137600

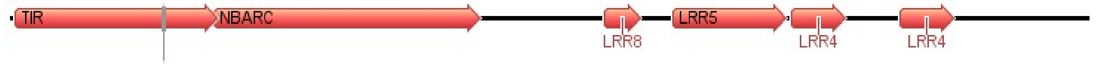

GLYMA_16G147000

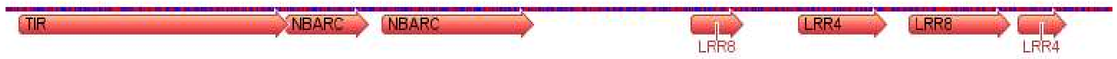

GLYMA_16G147400

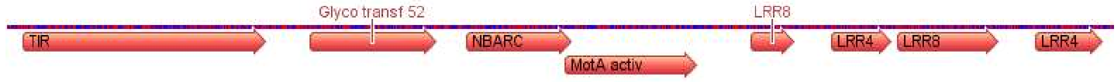

GLYMA_16G159100

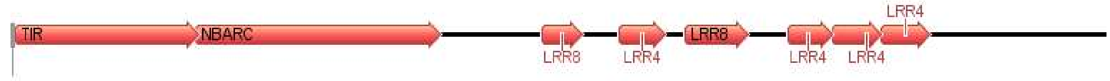

GLYMA_16G159200

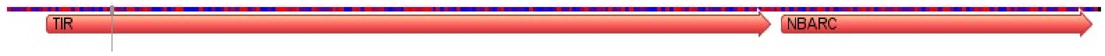

GLYMA_16G159400

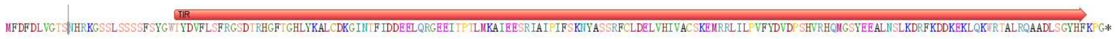

GLYMA_16G159600

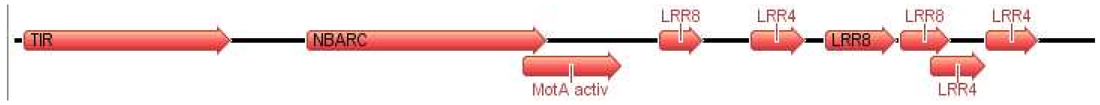

GLYMA_16G159700

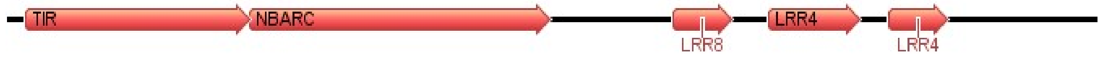

GLYMA_16G198900

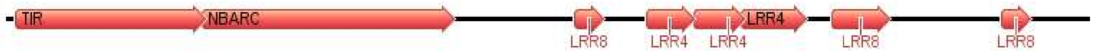

GLYMA_16G209000

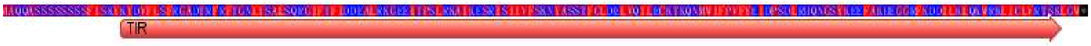

GLYMA_16G210600

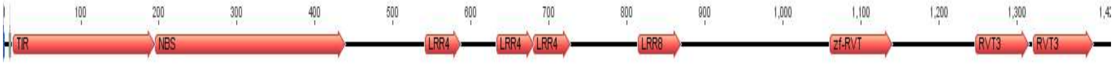

GLYMA_16G210800

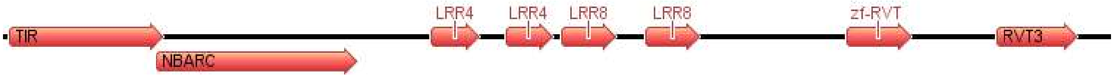

GLYMA_16G211400

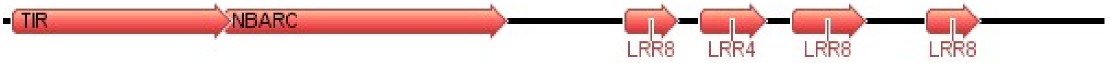

GLYMA_16G212300

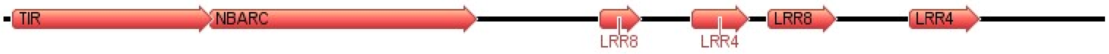

GLYMA_16G213700

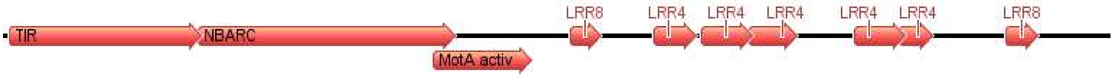

GLYMA_16G213800

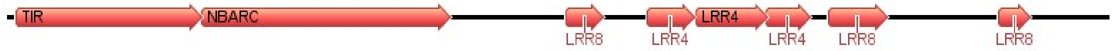

GLYMA_16G213900

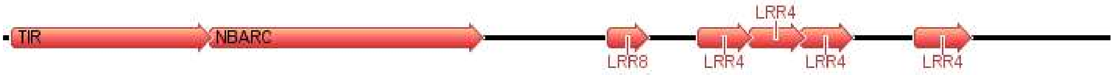

GLYMA_16G214000

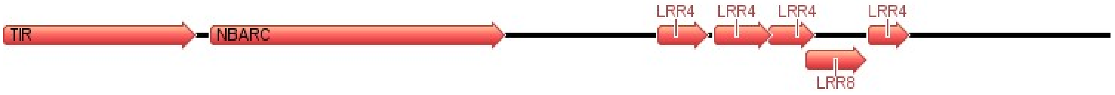

GLYMA_16G214100

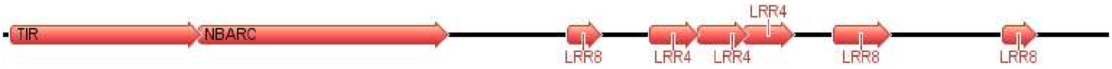

GLYMA_16G214200

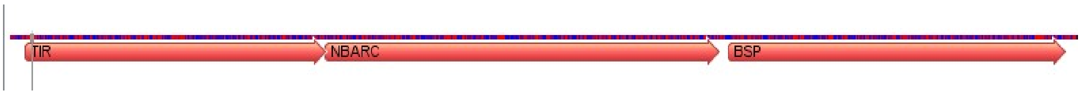

GLYMA_16G214300

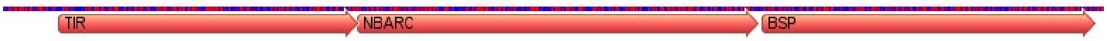

GLYMA_16G214500

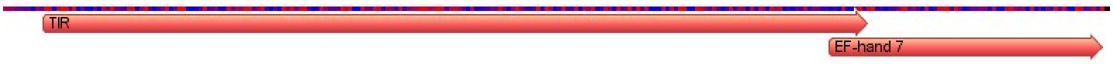

GLYMA_16G214600

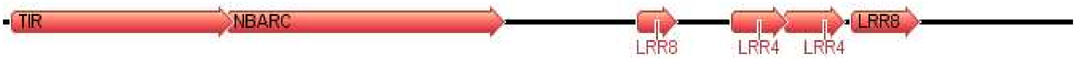

GLYMA_16G214700

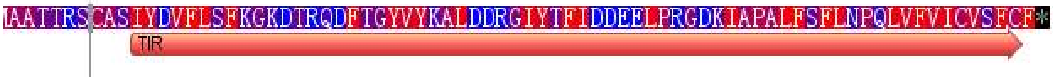

GLYMA_16G214800

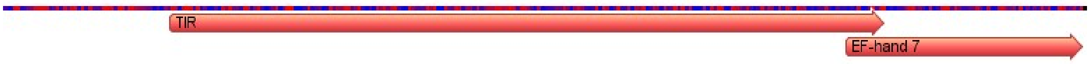

GLYMA_16G214900

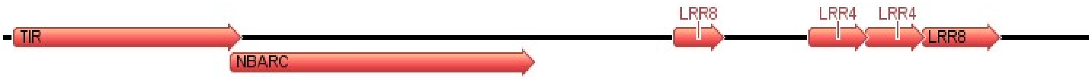

GLYMA_16G215000

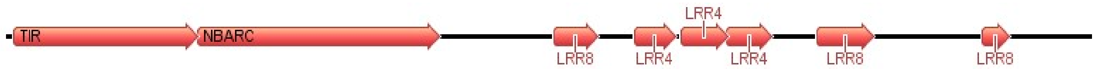

GLYMA_16G215100

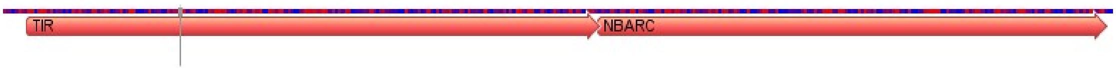

GLYMA_16G215200

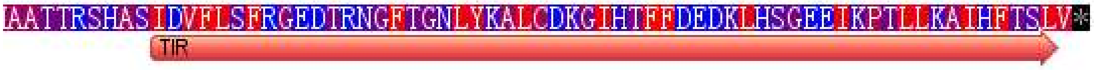

GLYMA_16G215400

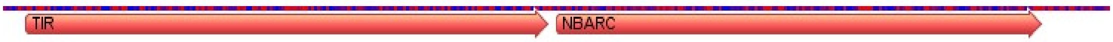

GLYMA_17G197100

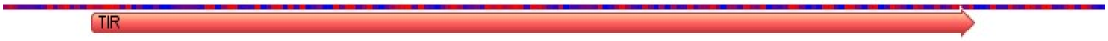

GLYMA_18G127800

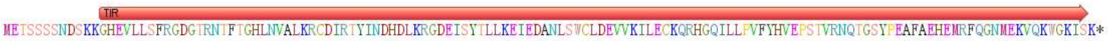

GLYMA_18G127900

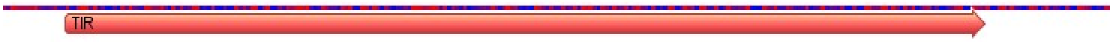

GLYMA_18G128000

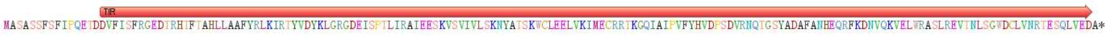

GLYMA_19G022700

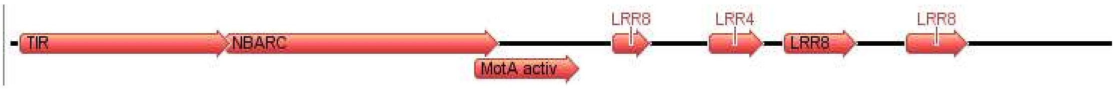

GLYMA_19G054700

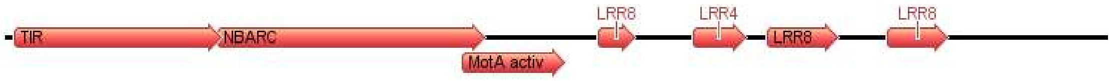

GLYMA_19G054800

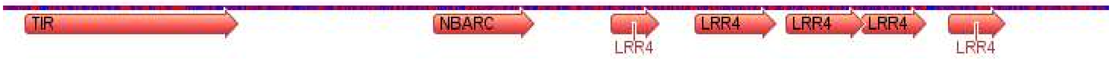

GLYMA_19G054900

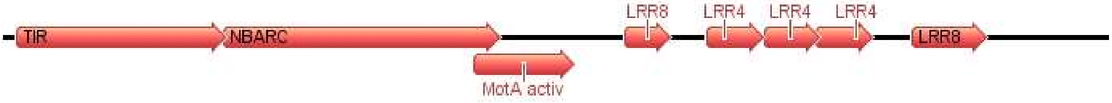

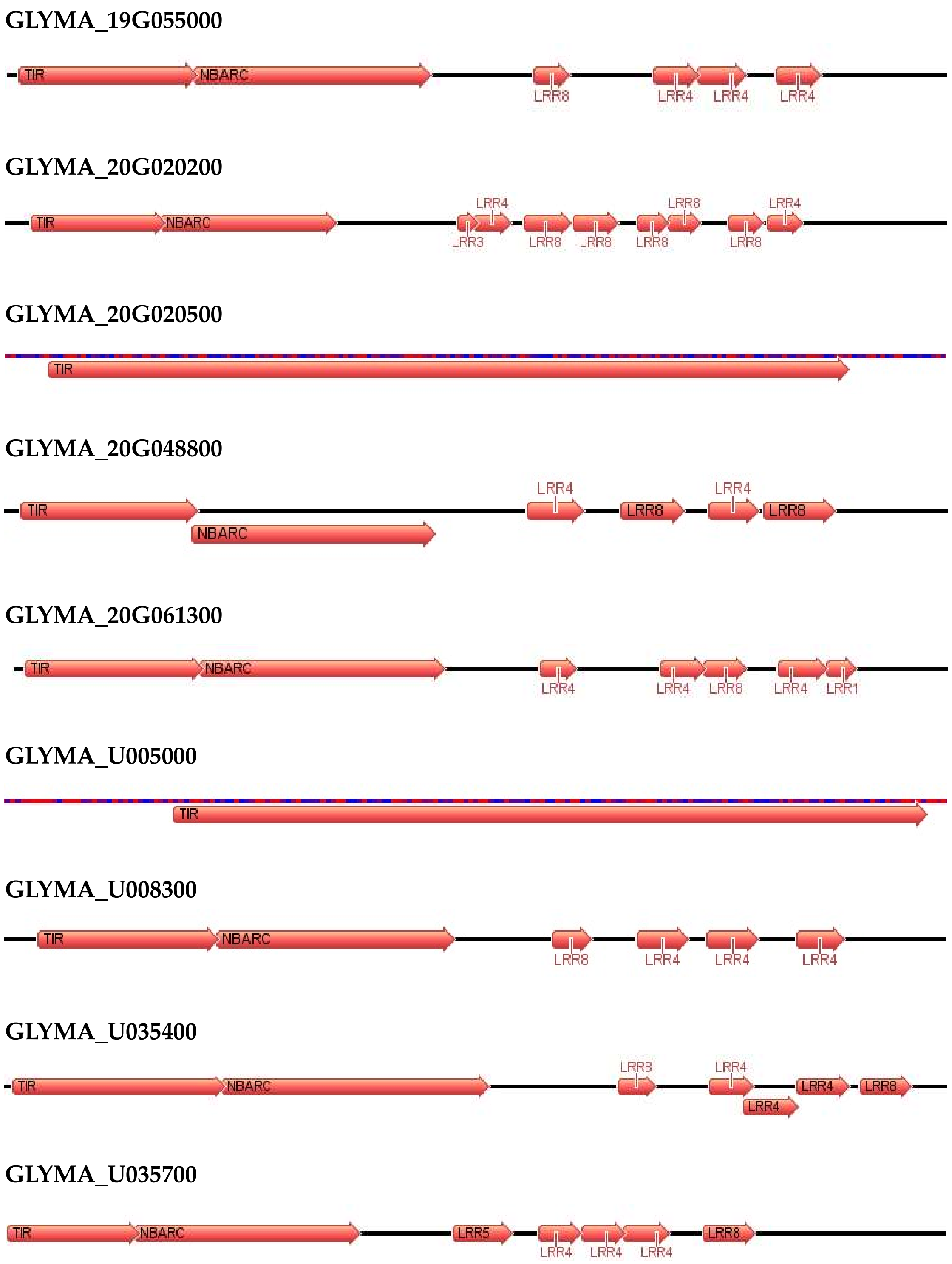

Figure S3. Domain diagram of 210 TNL-like proteins**.** Structural domain prediction and position calibration of the 210 TNL-like proteins using GENEIOUS v4.8.4 software based on PFAM database (http://pfam-legacy.xfam.org/) (accessed on 18 December 2020).

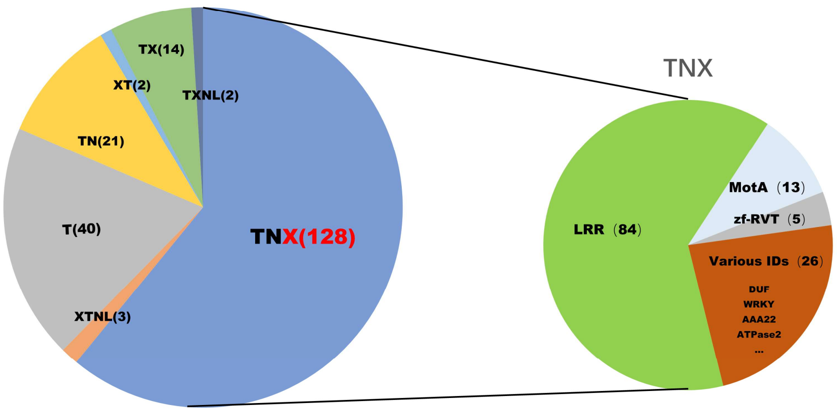

Figure S4. Classification of 210 soybean TNL-like proteins. The 210 soybean TNL-like proteins were grouped by domain type.

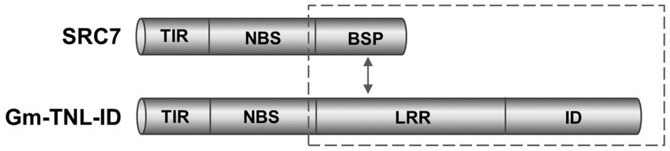

Figure S5. Domain architecture of SRC7 and soybean Gm-TNL-ID proteins. The above diagram shows the basic structural domains of SRC7 and the Gm-TNL-ID proteins we had screened.

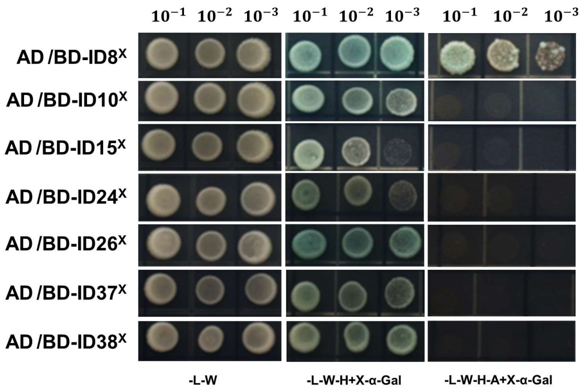

Figure S6. Evaluation of these BD-Gm-TNL-ID^X bait vectors. The auto-activation activity of these BD-Gm-TNL-ID^X were tested. The yeast cells containing indicated constructs were grown on different selective media, and 100 μl of X-α-Gal was added visualized the reporter expression. The yeast concentration gradients were 10^{-1},10^{-2} and 10^{-3}, respectively.

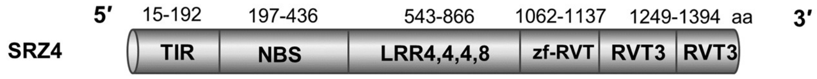

Figure S7. Domain architecture of SRZ4. Domain architecture of SRZ4. These grey boxes indicate TIR, NBS, LRR4, LRR4, LRR4, LRR8, zf-RVT, RVT3 and RVT3 domains, respectively.

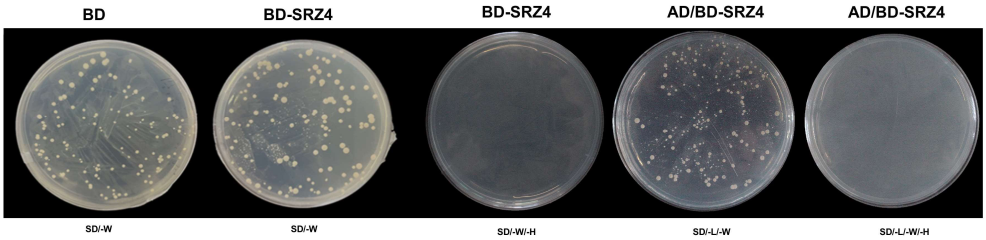

Figure S8. Testing the toxicity and auto-activation of SRZ4. For toxicity validation, the yeast cells containing BD-SRZ4 was grown on minimal media (SD/-W). For auto-activation activity validation, the yeast cells containing BD-SRZ4 and AD were grown on minimal media (SD/-L/-W) and stringent selective media (SD/-L/-W/-H).

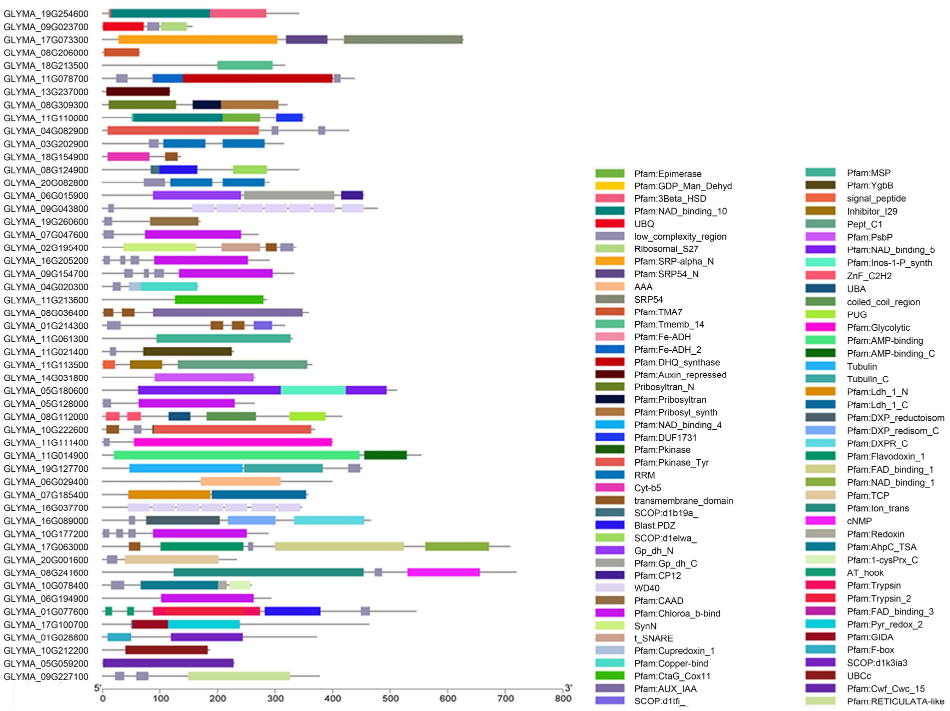

Figure S9. Structural domain prediction analysis of the soybean protein interacting with SRZ4. Schematic diagram of domain prediction for 48 soybean proteins interacting with SRZ4 using BATCH SMART in TBtools.

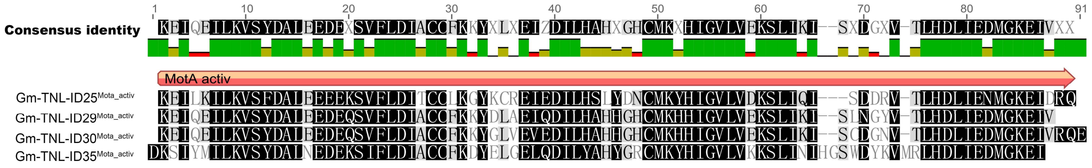

Figure S10. Sequence alignment of the MotA_activ domain included in four Gm-TNL-ID^{X} domains interacting with SMV component proteins. Sequence alignment of the MotA_activ domain included in four Gm-TNL-ID^{X} domains interacting with SMV component proteins using GENEIOUS v4.8.4 software.

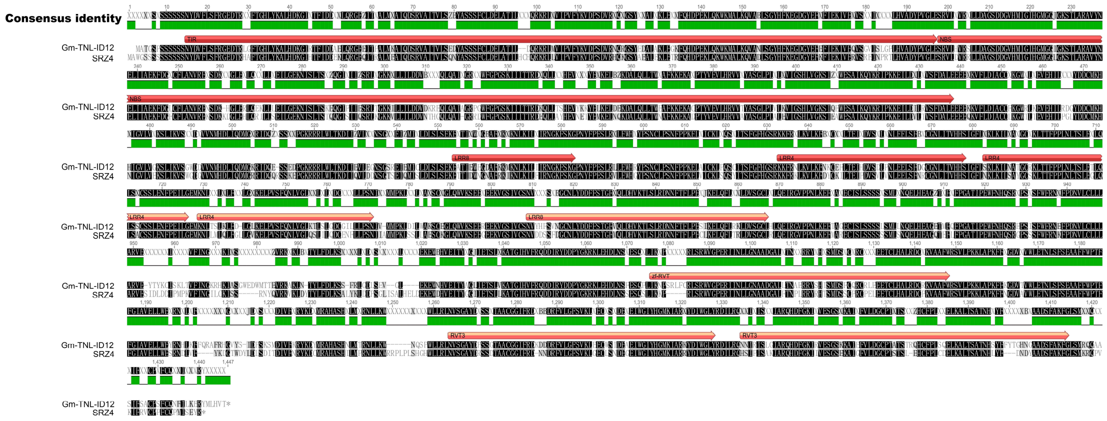

Figure S11. Sequence alignment of the Gm-TNL-ID12 and SRZ4. Sequence alignment of the Gm-TNL-ID12 and SRZ4 using GENEIOUS v4.8.4 software.
